# Supplementary material for: Automated preparation of plasma lipids, metabolites, and proteins for LC/MS-based analysis of a high-fat diet in mice
Source: J Lipid Res. 2024 Jul 25;65(9):100607. doi: 10.1016/j.jlr.2024.100607 (PMC11399584; doi:10.1016/j.jlr.2024.100607)
Supplement: VU-Supporting table titles-May2024 [file mmc1.docx]

### Table S1: Compounds extracted from polypropylene tubes vs. glass vials.

Table S2A: The reported CV of each lipid class was calculated from heavy labeled internal standards included in every sample in the accuracy and precision of the automated MTBE-PAL, using a formula CV =σ/mean(Peak Area); R^2^ was calculated based on the linear model of observed (Peak Area(light std)/Peak Area(heavy std)) ~ Theoretical ratio (light std/heavy std)

S2B: The reported CV of each metabolite was calculated from heavy labeled internal standards included in every sample in the accuracy and precision of the automated MTBE-PAL, using a formula CV =σ/mean(Peak Area) ; R^2^ was calculated based on the linear model of observed (Peak Area(light std)/Peak Area(heavy std)) ~ Theoretical ratio (light std/heavy std)

S2C: Mean log10 transformation of geometric mean (n=3) of compounds belong to the top 10 most abundant plasma metabolites and lipids per lipid class. Linear model was used to correlate measurement between two weeks for lipids and metabolites.

S2D: Carryover of top 10 most abundant plasma metabolites and lipids per class (%)

Table S3A: Concentration of lipids (μM) in 25 μL mouse plasma.

S3B: Regression results on plasma lipids associated with circadian rhythm effect.

S3C: Regression results on plasma lipids associated with age in mice.

S3D: Regression results on plasma lipids associated with HFD diet in mice.

Table S4A: MS measurement of metabolites in 25 μL mouse plasma.

S4B: Regression results on plasma metabolites associated with circadian rhythm effect.

S4C: Regression results on plasma metabolites associated with age in mice.

S4D: Regression results on plasma metabolites associated with HFD diet in mice.

Table S5A: Relative quantification of proteins in 25 μL mouse plasma.

S5B: Regression results on plasma proteins associated with circadian rhythm effect.

S5C: Regression results on plasma proteins associated with age in mice.

S5D: Regression results on plasma proteins associated with HFD diet in mice.

Table S6: Average total plasma lipid class concentrations calculated by summing each measured molecular species' concentration (concentration +/- SEM) (n=10).

Table S7: Total concentration (uM +/- SEM) of lipids containing ω-3 and ω-6 FA.
