# Supplementary material for: Automated preparation of plasma lipids, metabolites, and proteins for LC/MS-based analysis of a high-fat diet in mice
Source: J Lipid Res. 2024 Jul 25;65(9):100607. doi: 10.1016/j.jlr.2024.100607 (PMC11399584; doi:10.1016/j.jlr.2024.100607)
Supplement: Vu-Supporting figures-July2024 [file mmc2.docx]

### **SUPPLEMENTAL INFORMATION:**

**Automated preparation of plasma lipids, metabolites, and proteins for LC/MS-based analysis of a high-fat diet in mice**

Ngoc Vu^1^,Tobias M Maile^1^, Sudha Gollapudi^1^, Aleksandr Gaun^1^, Phillip Seitzer^1^, Jonathon J O’Brien^1^, Sean R Hackett^1^, Jose Zavala-Solorio^1^, Fiona E McAllister^1^, Ganesh Kolumam^1^, Rob Keyser^1^, Bryson D Bennett^1,^*

Calico Life Sciences LLC, South San Francisco, CA 94080, USA


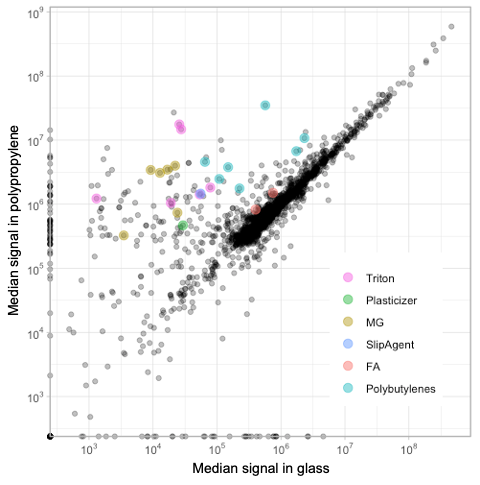


### Figure S1: Scatter plot of mass spectrometry features from samples manually extracted in glass vials vs. polypropylene tubes (n=3).

###

1. Metabolomics


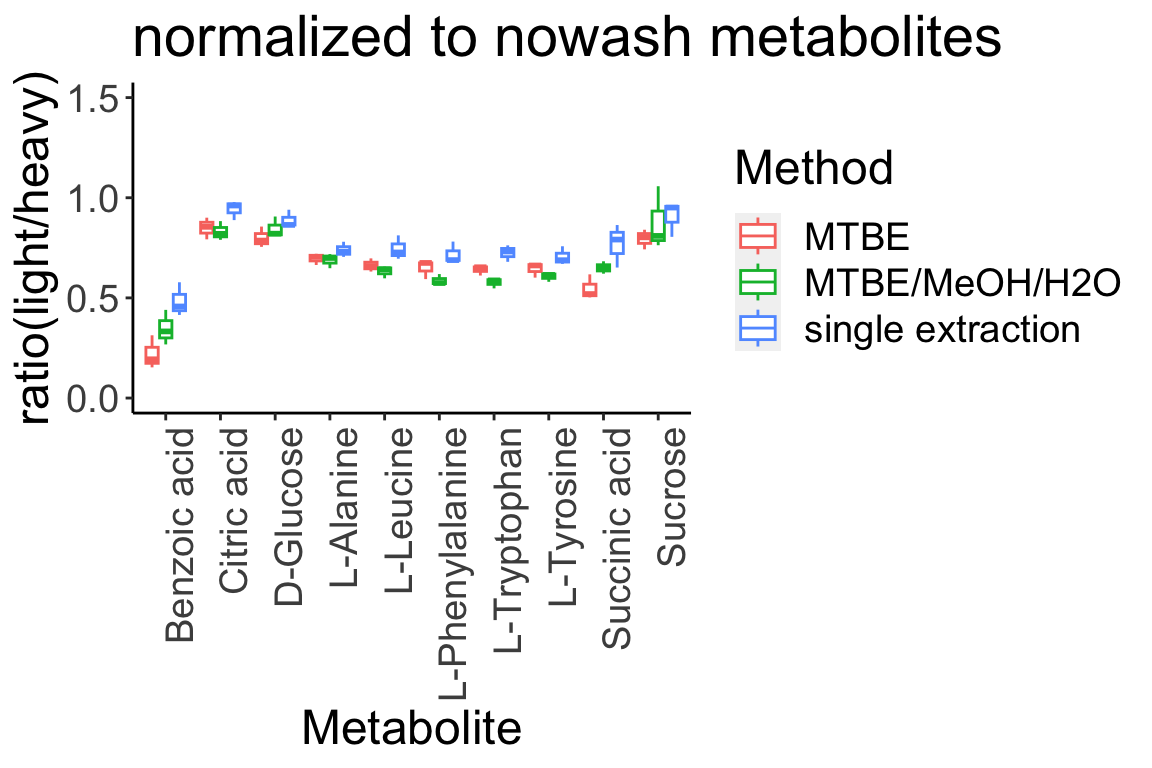


1. Lipidomics


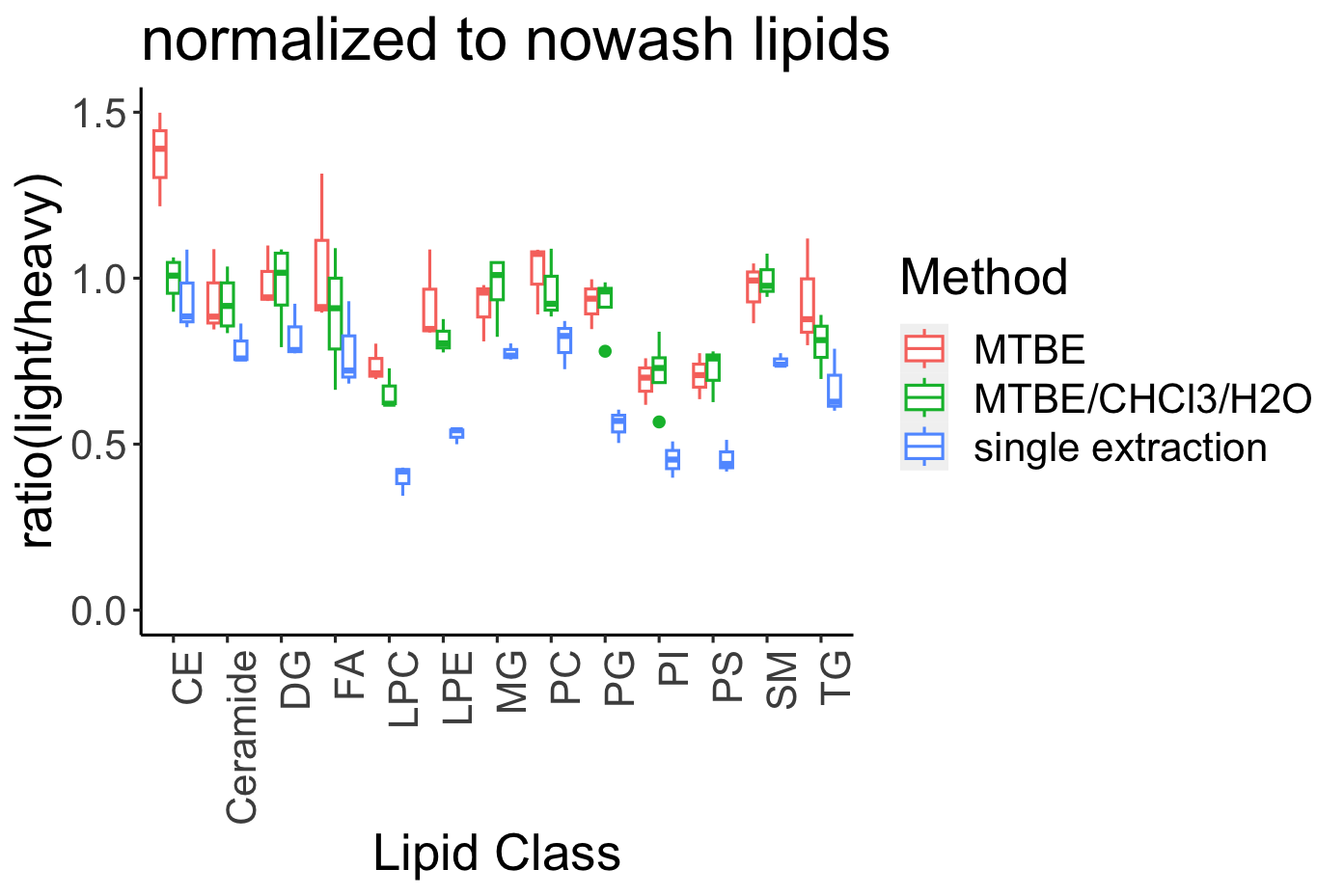


Figure S2: Recover efficiency of lipids and metabolites after LLE (n=3). Red= 100% MTBE, Green= MTBE/MeOH/H_2_O (3.33:1:1,v/v/v), Blue = without second extraction.

###
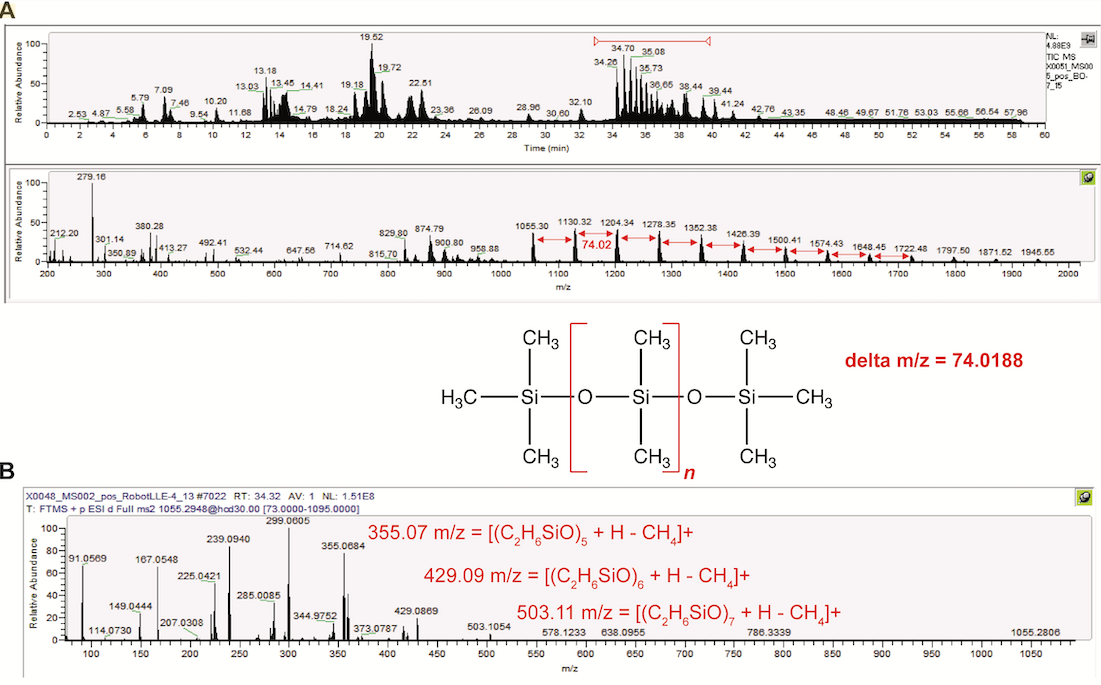


###
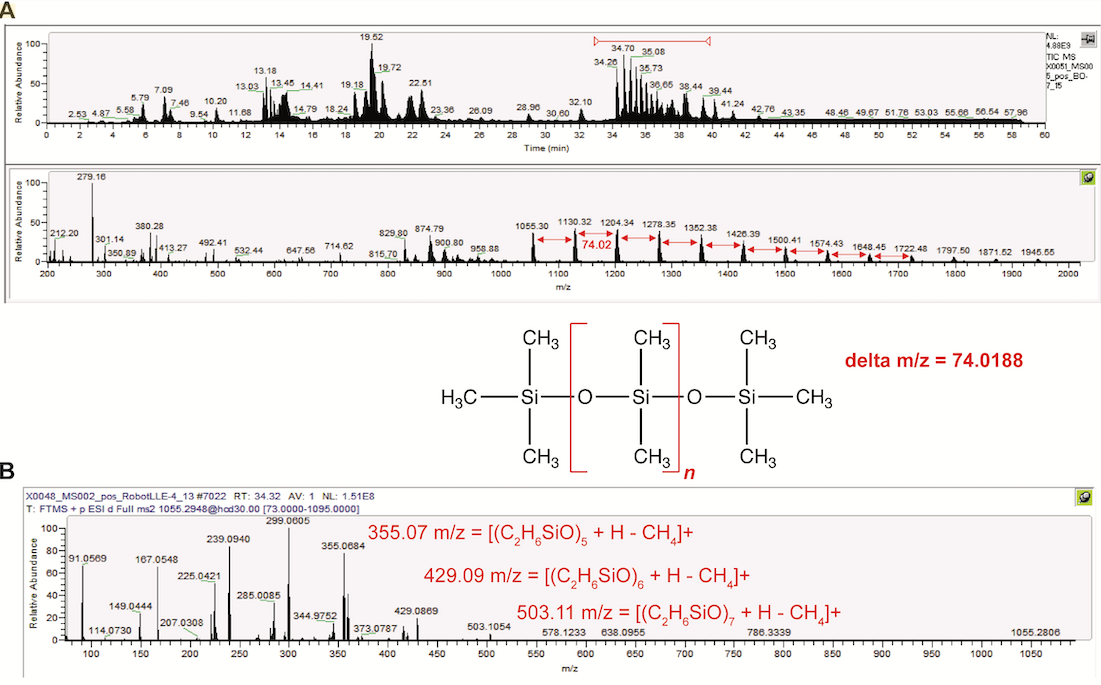


### Figure S3A: Polysiloxane EIC and MS/MS when using blue silicone cap - Top-MS1, bottom-MS2. Identification of polysiloxanes was done by matching their exact masses and the 74.02 D spacing between peaks in both the MS^1^, and the MS^2^ fragments of each precursor.


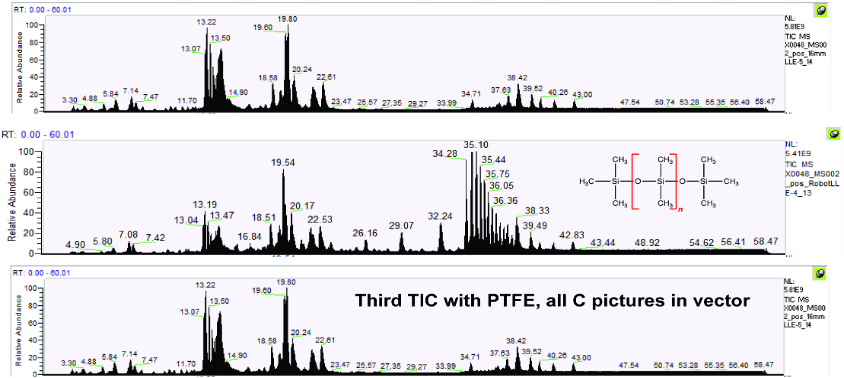


### Figure S3B: MS1 spectra of lipid data with PTFE disc (Pure_PTFE) (top) and silicon cap (bottom)


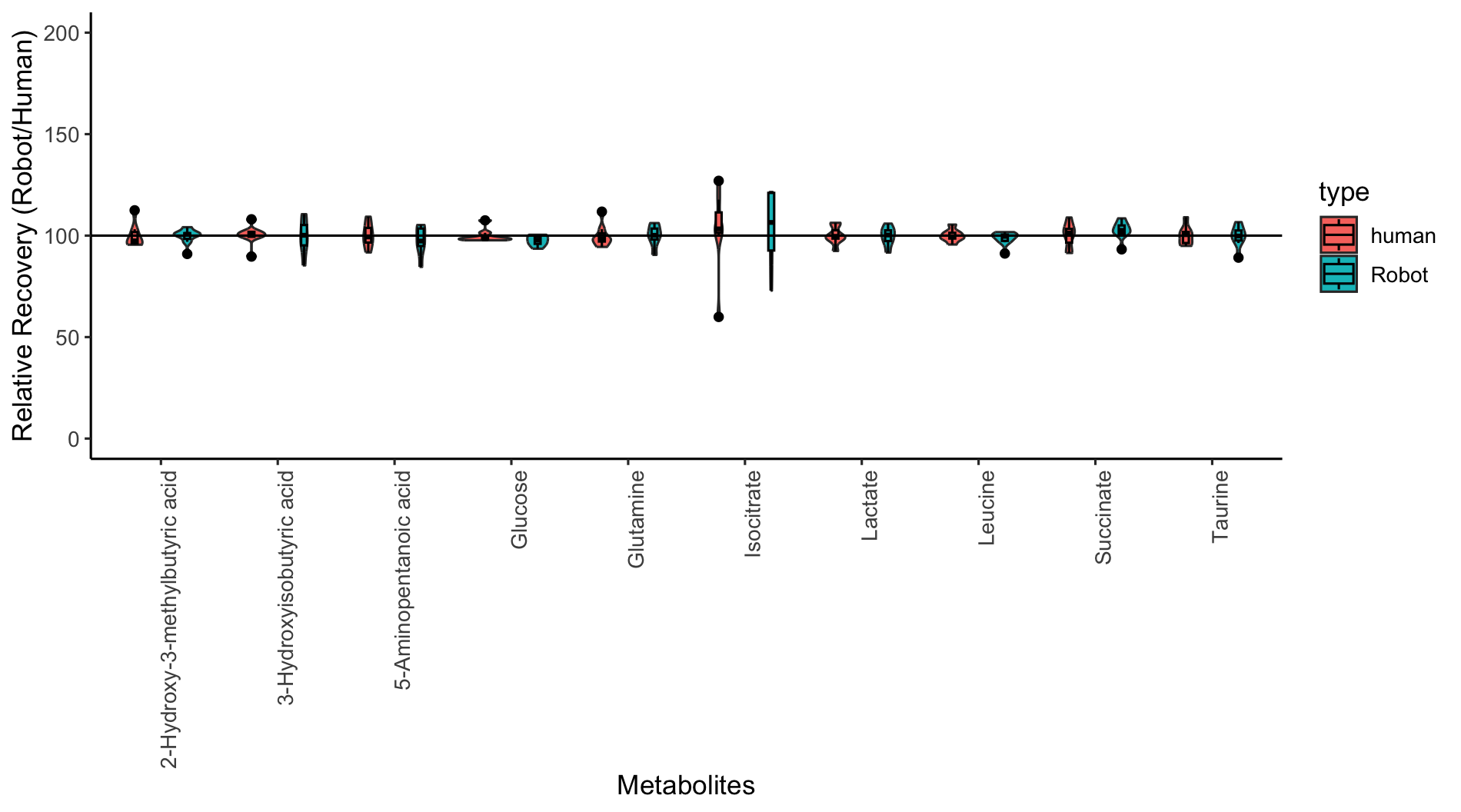


Figure S4A: Relative recovery of the 10 plasma metabolites with the highest intensity. 10μL of mouse plasma was extracted manually (n=10) or by robot PAL (n=7). PeakAreaTop of each plasma metabolite extracted by the robot was normalized to the mean of PeakAreaTop of the manual extractions.


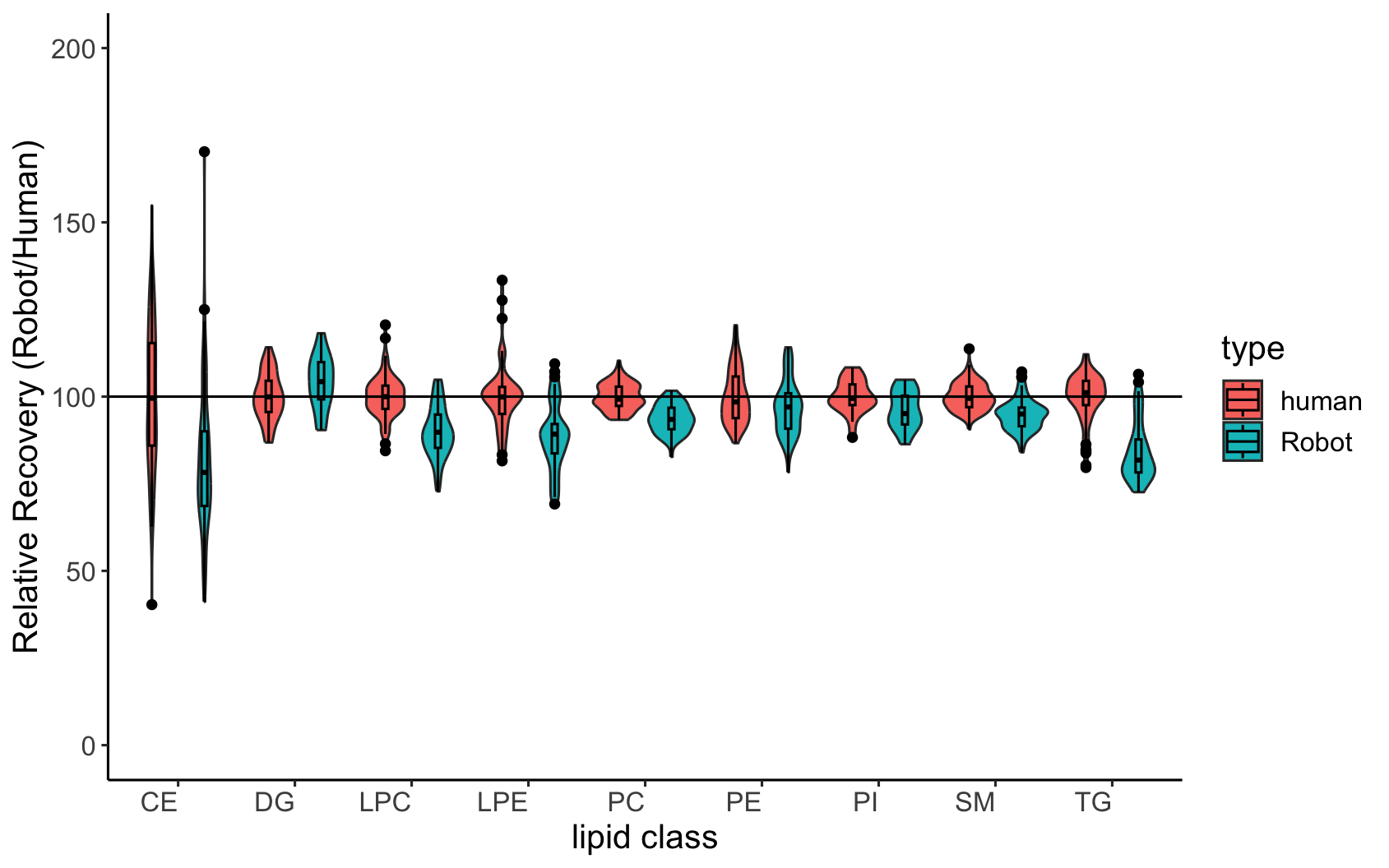


Figure S4B: Percent recovery of Top 10 plasma lipids per lipid class. 10μL of mouse plasma was extracted manually (n=10) or by robot PAL (n=7). LipidSplash standards were spiked in post-LLE, during resuspension. Peaks were normalized to the class-specific LipidSplash standard. Relative recovery represents the concentration of lipids relative to the mean of the manually extracted lipids from that class.


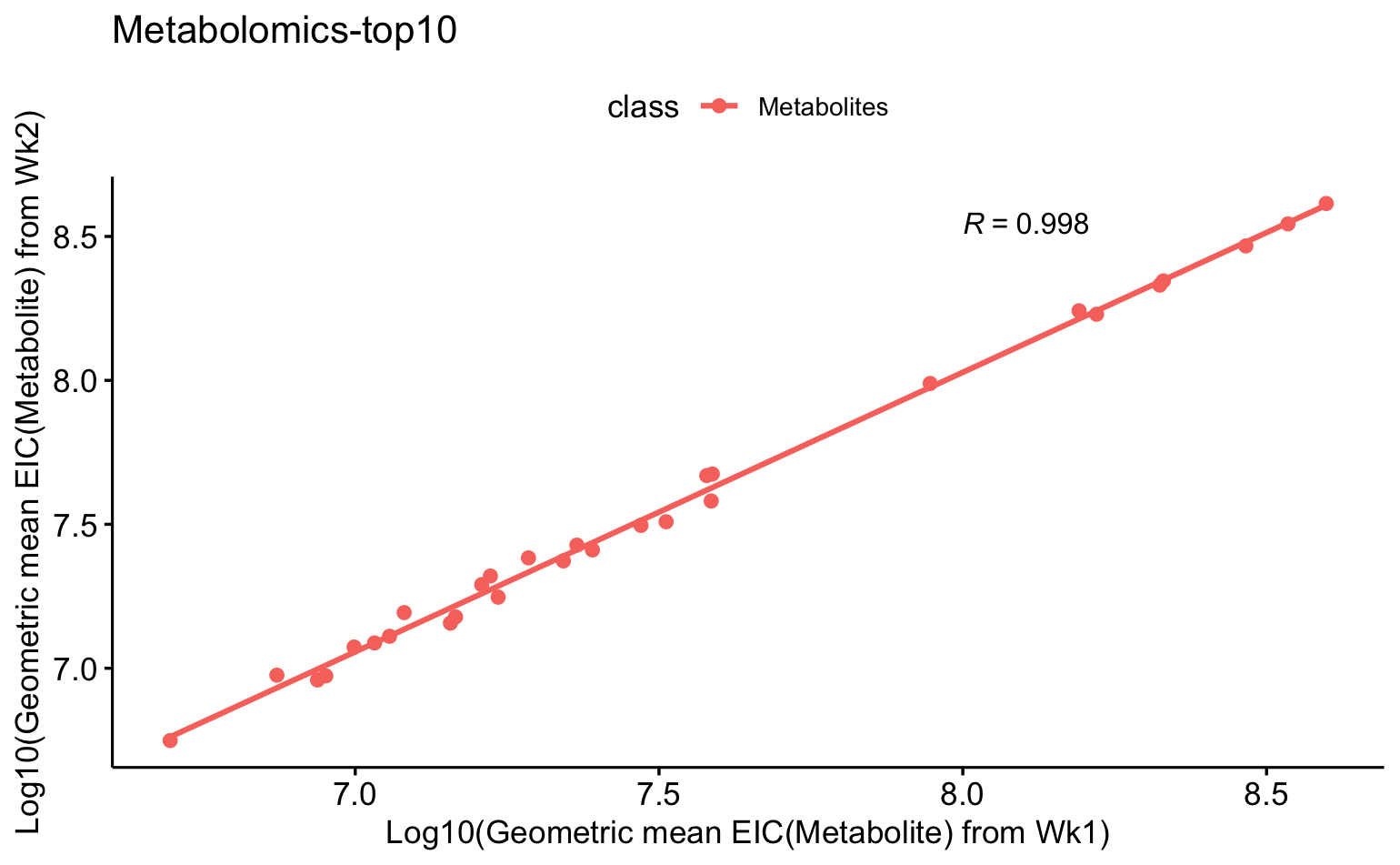


Figure S4C: The correlation between the top 10 most abundant plasma metabolites (20, 30, and 50 μL plasma, n = 3) is represented in a scatter plot. The x-axis denotes the natural logarithm of the geometric mean of peak area obtained in week 1, while the y-axis represents the natural logarithm of the geometric mean of peak area collected in week 2. Each dot on the plot corresponds to the mean log-transformed peak area of a specific metabolite per unit of plasma volume.


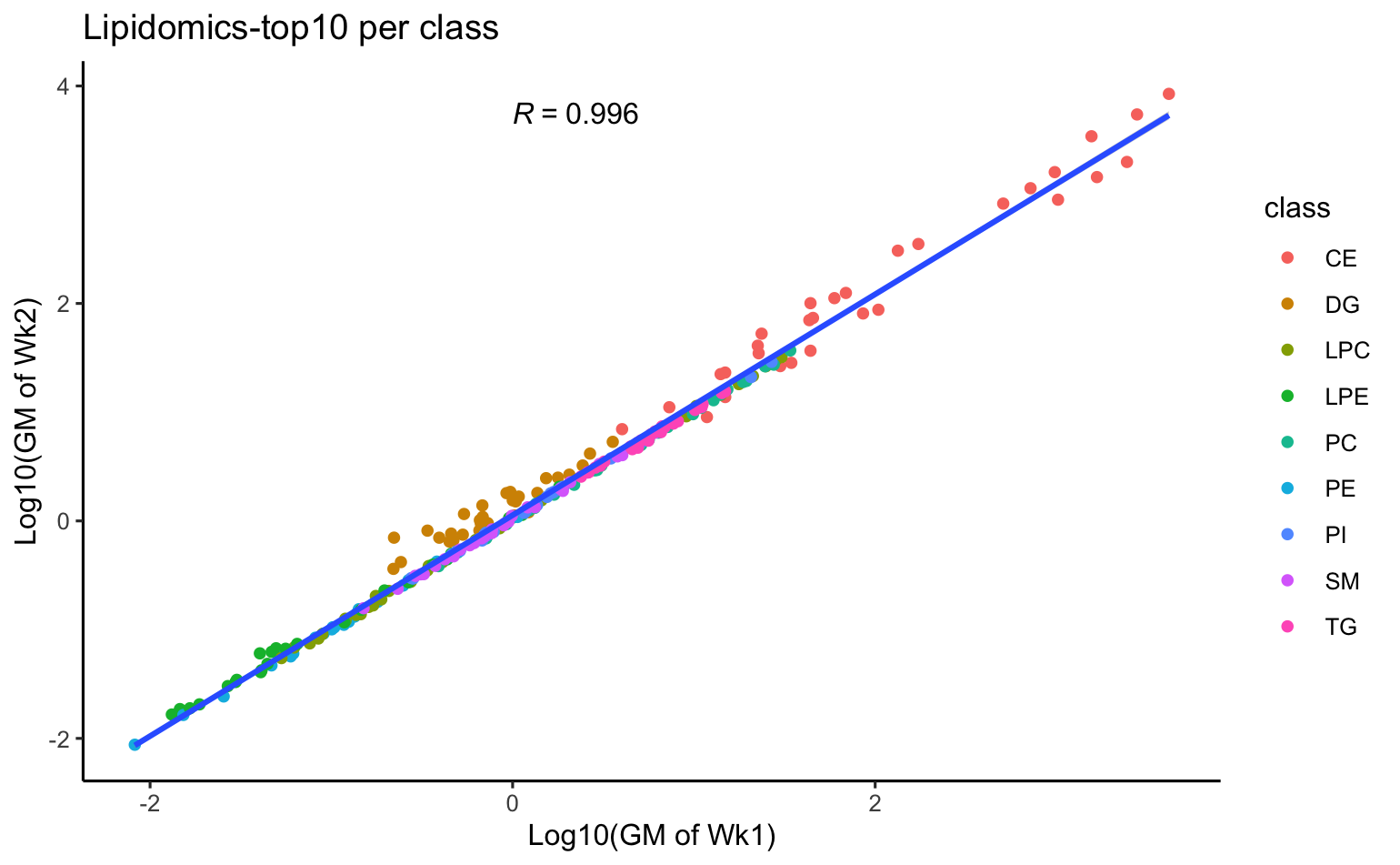


Figure S4D: Correlation between the top 10 most abundant plasma lipids from each lipid class, as extracted using automated liquid-liquid extraction (LLE) from three samples of plasma volumes (20, 30, and 50 μL). The peak areas of each analyte were normalized to the peak area of the corresponding LipidSplash standard, followed by natural logarithm transformation. The x-axis represents the natural logarithm of the geometric mean of lipid concentration (nM) obtained in week 1, and the y-axis represents the natural logarithm of the geometric mean of lipid concentration (nM) obtained in week 2. Each dot represents the average log-transformed quantification of unique lipids per plasma volume. The lipid classes are represented by different colors.


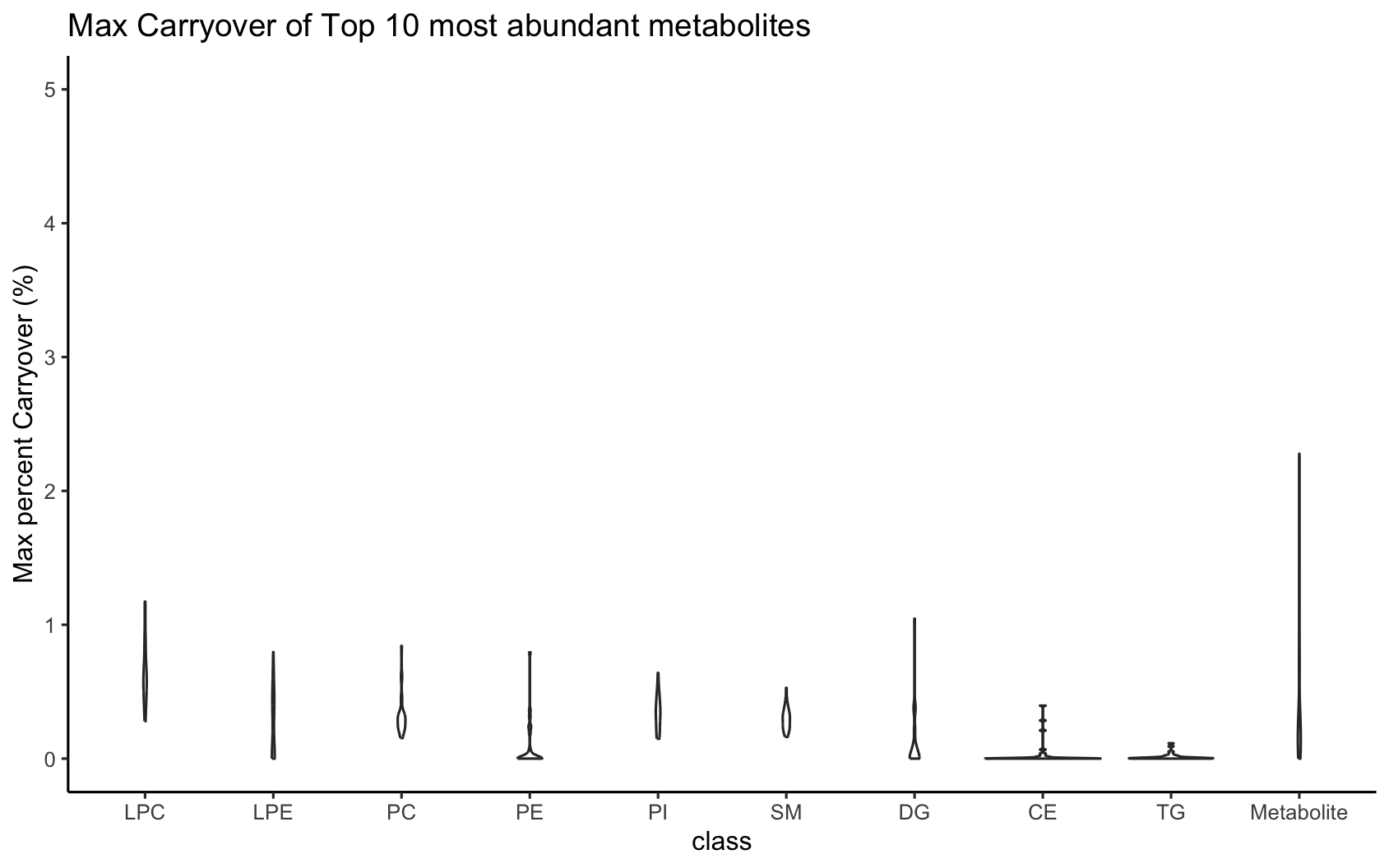


Figure S4E: Percent carry over of top 10 most abundant plasma metabolites and lipids per lipid classes. Samples were arranged and extracted with a blank in between each plasma sample (n=3) at various plasma volumes (20, 30 and 50 μL). The carry over for metabolites and lipids was calculated as the fraction of the peak area of the analyte in the blank compared to the plasma injected before it.


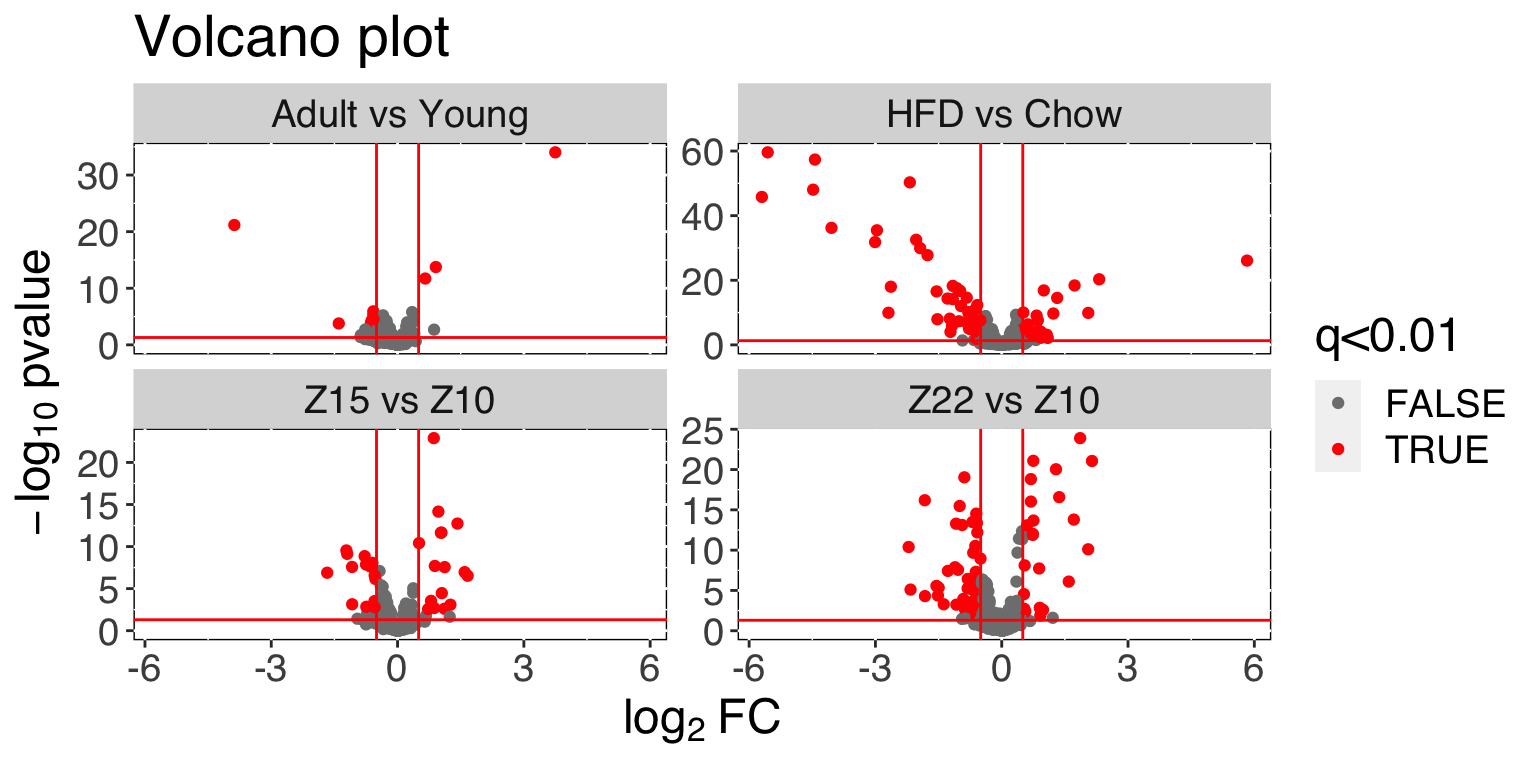


Figure S5A: Significant metabolites changed with time point, age, and diet (q<0.01)


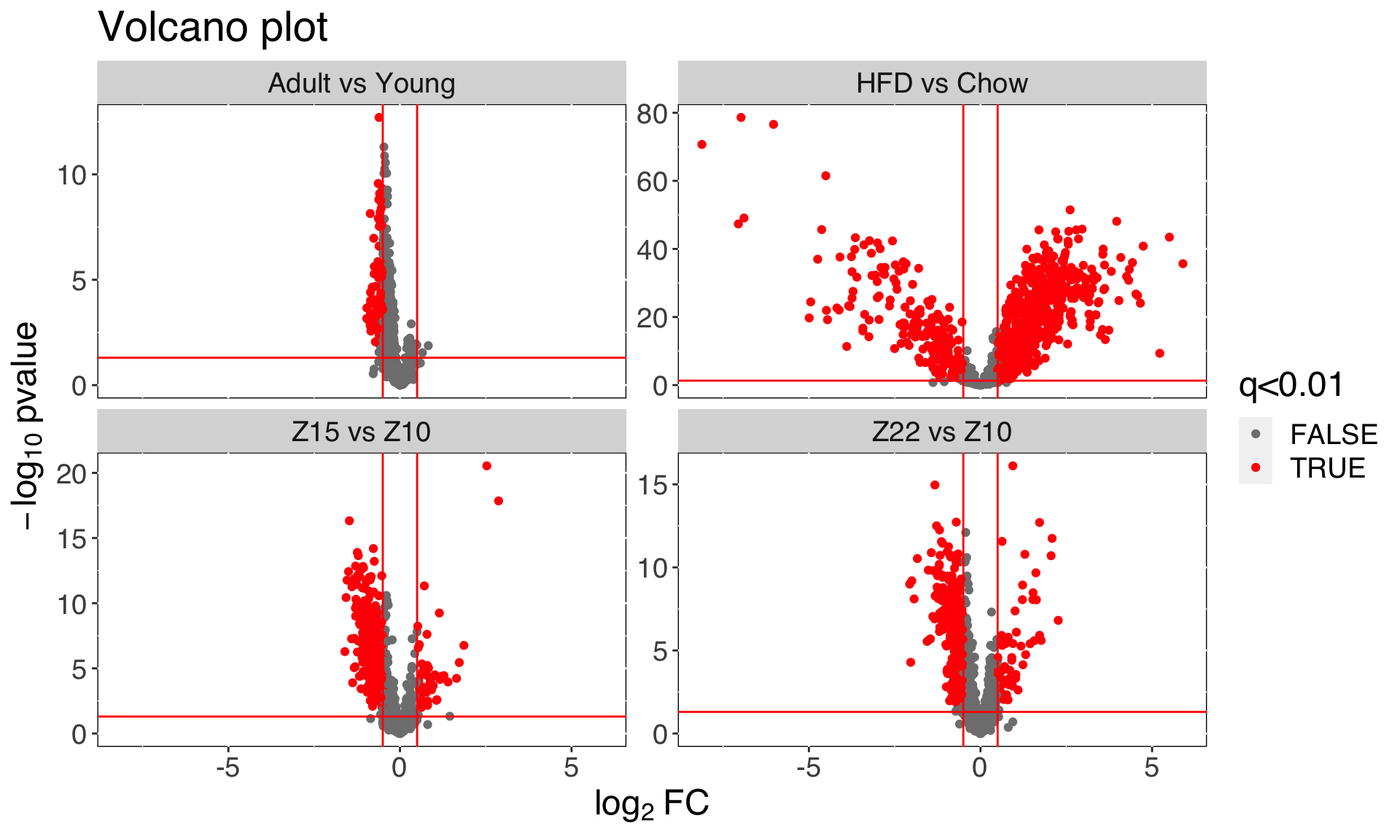


Figure S5B: Significant lipids changed with time point, age, and diet (q<0.01)


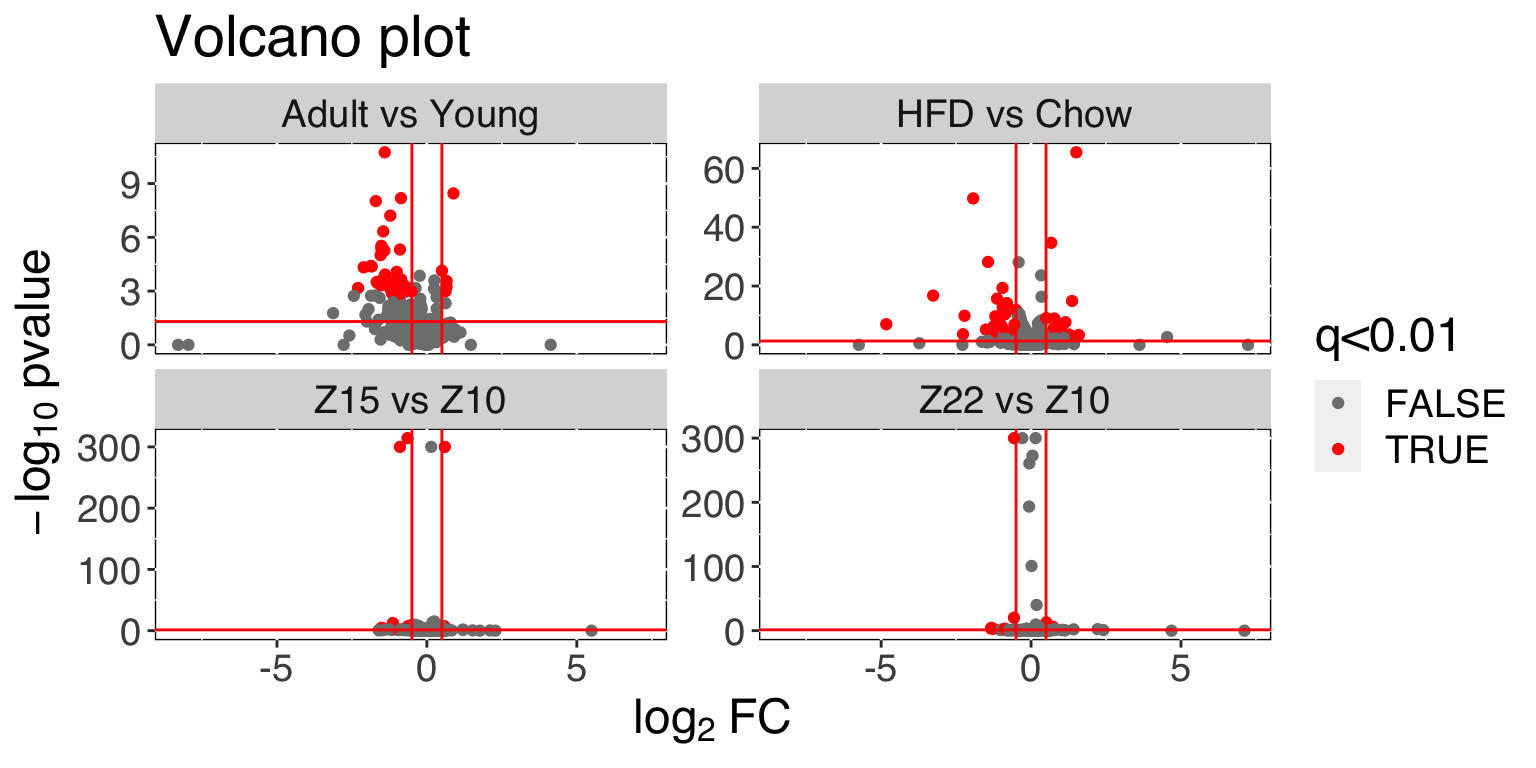


Figure S5C: Significant proteins changed with time point, age and diet (q<0.01)


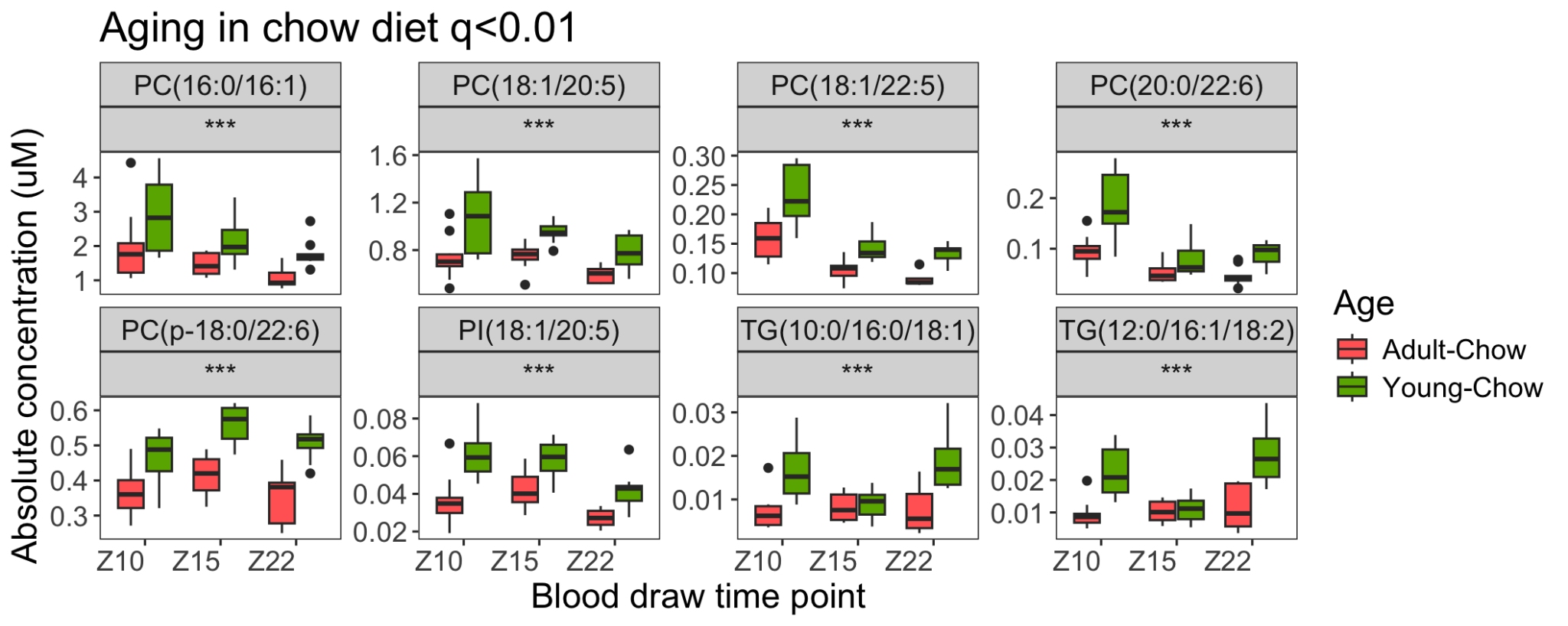


Figure 6A: Selected lipids showing a significant age effect (*** p-value <0.001, * p-value <0.05) between the young-chow (green boxes) and adult-chow (red boxes) groups.


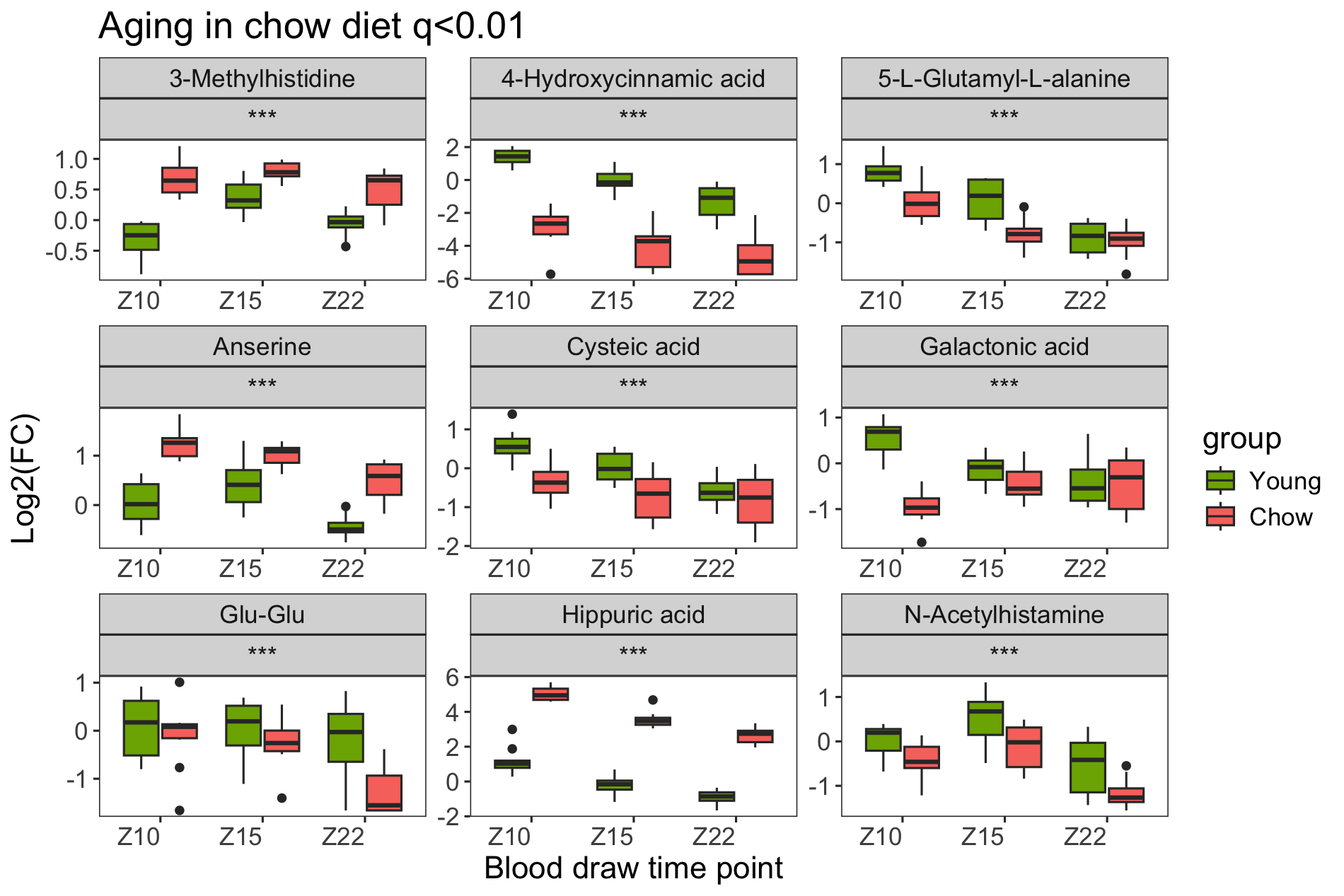


Figure S6B: Selected metabolites showing a significant age effect (*** p-value <0.001, * p-value <0.05) between the young-chow (green boxes) and adult-chow (red boxes) groups. Value are normalized the the mean value in adult-chow at Z10.


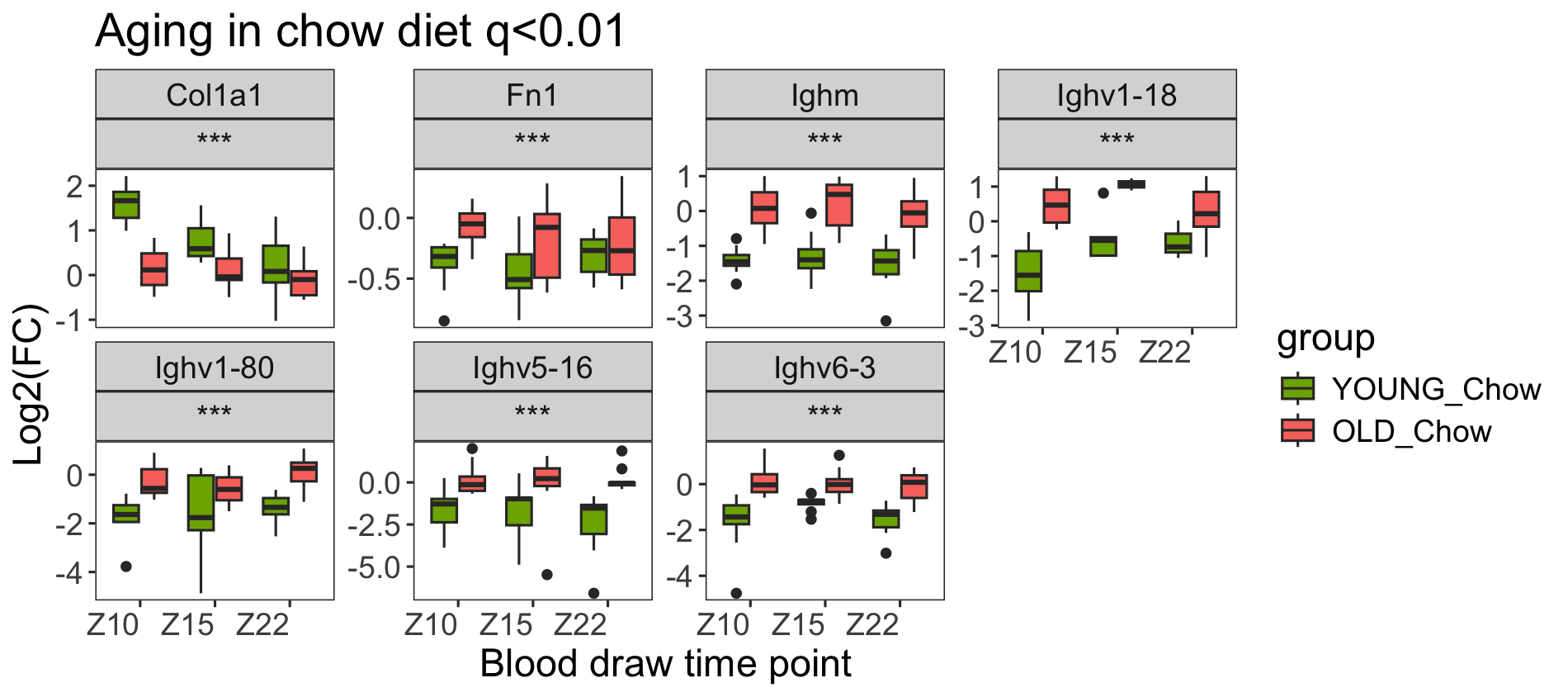


Figure S6C: Selected proteins showing a significant age effect age effect (q<0.01,*** p-value <0.001, * p-value <0.05) between the young-chow (green boxes) and adult-chow (red boxes) groups. . Proteins were normalized to the bridge included in every plex.


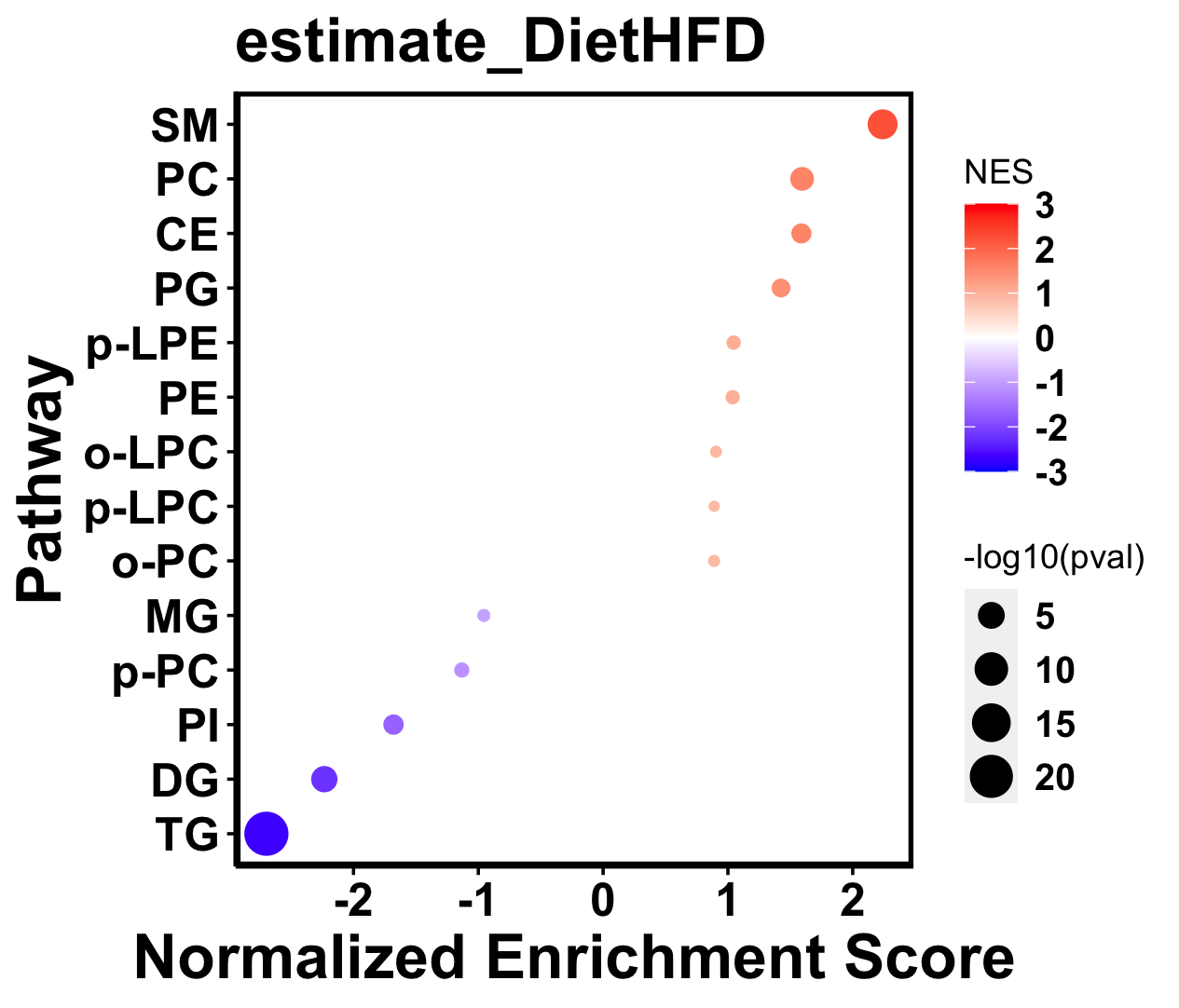


Figure S7A: Lipid Class enrichment in adult mice under HFD. Color scale reflects the log2 transformation of the regression coefficient of the term HFD. The size of circle is -log10(pvalue).


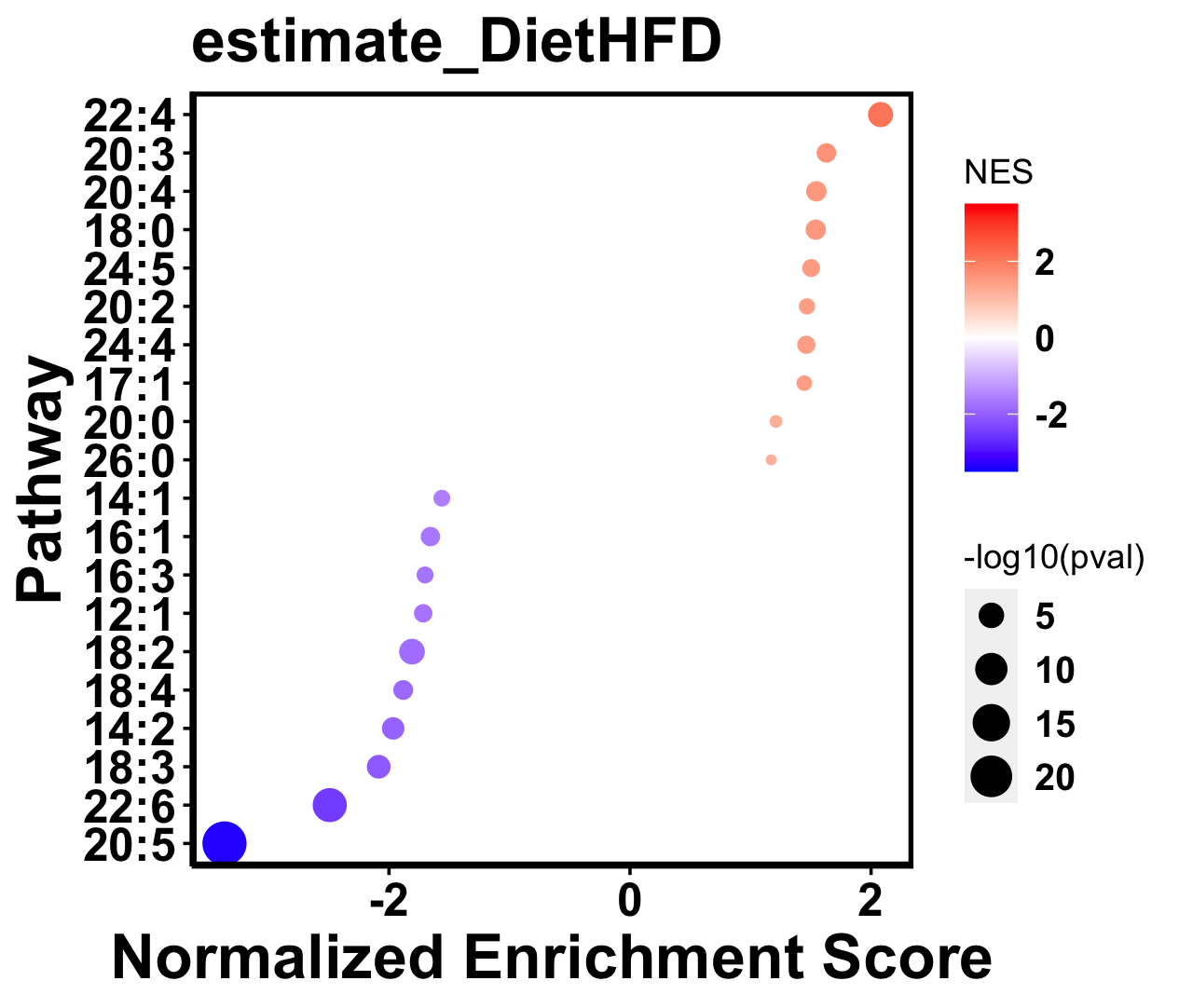


### Figure S7B: Enrichment of acyl chain composition of lipids in adult mice under HFD
