# Supplementary material for: Automated preparation of plasma lipids, metabolites, and proteins for LC/MS-based analysis of a high-fat diet in mice
Source: J Lipid Res. 2024 Jul 25;65(9):100607. doi: 10.1016/j.jlr.2024.100607 (PMC11399584; doi:10.1016/j.jlr.2024.100607)
Supplement: Custom Components on the PAL [file mmc10.docx]

The PAL system contains the following customized parts and consumables:

- Custom vials

SureStop 2 mL vials (#C5000-1W) with National Scientific custom magnetic caps (#CPSH0003) from Thermo Scientific for MTBE-LLE extraction

PTFE septa (TFC5000-52AE) from National Scientific for fraction storage during automated extraction using PAL.

- Syringes

Dilutor tool needle: The diluter needle was changed to a Hamilton 23 gauge side-port needle (P/N 7732-06).

Syringes on both heads: Syringes on both PAL heads were changed to 1 mL Hamilton gastight syringes (P/N 1000) with 23 gauge side-portneedles (P/N 7732-06)

- Reagent chiller assembly

We designed a custom chiller adapter for the MeOH/water reagent bottles to be chilled down to -20C. The aluminum adapter is placed inside a Mecour chiller, and the bottle is placed inside to keep the solvent chilled to -20C.

- Glass bottles for solvents

Glass bottles for MTBE and MeOH/water were outfitted with caps containing two solvent line ports. An output solvent line is attached to one port, and the other is loosely covered with a sticker. This allows vapor to vent and allows solvent to flow out of the bottle without hindrance due to vapor pressure.

- Aluminum vial racks

Calico designed custom 54-vial racks in aluminum to keep vials cold in the chilled peltier stack on the PAL. The PAL’s default racks were made of plastic, which did not stay cold through the many assay steps that involve opening and closing the peltier stack drawers.

- Nitrogen evaporator

Nitrogen evaporator of the 54 vials format (P/N 2305) was purchased from Analytical Sale and Services (Flander, NJ, USA). Vials was placed to top of the cooling plate (P/N CB-BVO) from Mecour (Groveland, MA, USA), connected to the chiller (P/N AD07R-40-S11B) purchased from PolyScience (Niles, IL, USA)

- Custom enclosure

Calico designed a custom enclosure for the PAL with a negative pressure vacuum vent to prevent solvent vapors from escaping into the lab.
