# Supplementary material for: Automated preparation of plasma lipids, metabolites, and proteins for LC/MS-based analysis of a high-fat diet in mice
Source: J Lipid Res. 2024 Jul 25;65(9):100607. doi: 10.1016/j.jlr.2024.100607 (PMC11399584; doi:10.1016/j.jlr.2024.100607)
Supplement: Outline_for_automated_PAL-LLE [file mmc11.pdf]

Chronos Method (unnamed Method)

Columns:

Number	Name	Token	Default value
1	NumSampleVials (even num only!)	%NumVials%	8
2	UpdateSolventModuleToFull	%YNFullSolvMod%	True
3	LogFileCounter	%YNCounterLog%	True
4	LogFileName	%FILENAME1%	{DateTime} LogFile.txt
5	LogFileName_Test	%FILENAME%	{DateTime} LogFile.txt
6	RuntimeLog	%RUNLOGFILENAME%	{DateTime} RunLogFile.txt
7	Source Tray Samples	%SRCTRAY%	Drawer 1:Slot1:1
8	Dest Tray Vials	%DESTTRAY%	Drawer 1:Slot1:1
9	DestA Tray	%TRAYA%	Drawer 2:Slot1:1
10	DestB Tray	%TRAYB%	Drawer 3:Slot1:1
11	Sample Volume [µL]	%SmplVol%	10
12	Dilutor Volume [µL]	%DilVol%	750
13	Dilutor Prime	%DilPrime%	False
14	DilutorPrime Volume [µL]	%DilPrimeVol%	200
15	MTBE Add1 Volume [µL]	%MTBE1Vol%	750
16	MTBE Add2 Volume [µL]	%MTBE2Vol%	600
17	MTBE Min Solv Mod [µL]	%MTBEMinSolvMod%	30000
18	DestA 1of2 Volume [µL]	%DestA1Vol%	520
19	DestA 2of2 Volume [µL]	%DestA2Vol%	860
20	DestB Volume [µL]	%DestBVol%	425
21	Incubation Time1 [min]	%INCTIME1%	5
22	Incubation Time2 [min]	%INCTIME2%	1
23	VialDisp Penetration Depth [mm]	%VialDispPenDepth%	20
24	VialDisp Penetration Depth MeOH [mm]	%VialDispPenDepthMeOH%	12
25	VialAsp Penetration Depth [mm]	%VialAspPenDepth%	10
26	TopLayerAsp Penetration Depth [mm]	%TopAspPenDepth%	23.5
27	TopLayerAsp Penetration Depth2 [mm]	%TopAspPenDepth2%	25

28	BottomLayerAsp Penetration Depth [mm]	%BotAspPenDepth%	33.6
29	Vortex Time 1st [sec]	%VRTXTIME1%	70
30	Vortex Time 2nd [sec]	%VRTXTIME2%	30
31	Vortex Speed [RPM]	%VRTXSPEED%	2000
32	Centrifuge Time [min]	%CENTTIME%	1
33	Centrifuge Speed [RPM]	%CENTSPEED%	5000
34	Post Clean With Solvent 1	%PostCleanSolvent1%	1
35	Post Clean With Solvent 2	%PostCleanSolvent2%	1
36	Enable Additional Prep1 0 or 1	%ADDPREP1%	1
37	***Hardware Settings ***	******************	**************
38	Autosampler_Left	%L_AUTOSAMPLER%	PAL:LeftHead
39	Autosampler_Right	%R_AUTOSAMPLER%	PAL:RightHead
40	Tool_Dilutor	%TOOL_DL%	DIL 1
41	Tool_100uL_Right	%TOOL_100R%	LS 3
42	Tool_1mL_Right	%TOOL_1mLR%	LS 2
43	Tool_1mL_Left	%TOOL_1mLL%	LS 1
44	Fill Speed 100uLSYR [µL/ s]	%FillSpeed100%	20
45	Fill Speed 1,000uLSYR [µL/ s]	%FillSpeed1000%	13
46	Fill Speed 1,000uLSYR_AQ [µL/ s]	%AQFillSpeed1000%	30
47	Fill Speed 1,000uLSYR_MTBE [µL/ s]	%MTBEFillSpeed1000%	50
48	Sample Fill Strokes	%SmplFillStrokes%	0
49	MTBEWash	%MTBEWash%	80
50	MeH2OWash	%MeH2OWash%	100
51	MTBE Fill Strokes	%MTBEFillStrokes%	1
52	Vortex Wash Delay [sec]	%VRTXWSHDEL%	35

Tasks:

1: SetRuntimeLogfile

Logfile: C:\Users\PAL-tower\Documents\Chronos\RunLogs\%RUNLOGFILENAME%

2: SolventModuleSelector

Set volume to Full for all three MTBE solvent reservoirs

Enabled:	| IF %YNFullSolvMod%=True THEN Return True ELSE Return False |
Runtime:	1
SolventModuleVolume:	100000 µL
MinimumSolventVolume:	%MTBEMinSolvMod% µL
VolumeUsed:	0 µL
SolventModuleAction:	RefillAll

3: WriteToLogfile

start new MTBE solvent dispense log file

Enabled:	| IF %YNFullSolvMod%=True THEN Return True ELSE Return False |
Runtime:	1
Logfile:	C:\Users\PAL-tower\Documents\Chronos\RunLogs\%FILENAME%
Text:	start new log file
IncludeTimeStamp:	True
Append:	| IF %YNFullSolvMod%=True THEN Return False ELSE Return True |

4: WriteToLogfile

counter logging

Enabled:	| IF %YNCounterLog%=True THEN Return True ELSE Return False |
Runtime:	1
Logfile:	C:\Users\PAL-tower\Documents\Chronos\RunLogs\CounterLogging.txt
Text:	start new counter logging file
IncludeTimeStamp:	True
Append:	False

5: RinseWashLiner

FW1:1, 5x

Enabled:	True
Runtime:	1
Autosampler:	PAL:LeftHead
WashLiner:	Fast Wash 1:1
Cycles:	5

6: RinseWashLiner

FW1:2, 5x

Enabled:	True
Runtime:	1
Autosampler:	PAL:LeftHead
WashLiner:	Fast Wash 1:2
Cycles:	5

7: RinseWashLiner

FW2:1, 5x

Enabled:	True
Runtime:	1
Autosampler:	PAL:LeftHead
WashLiner:	Fast Wash 2:1
Cycles:	5

8: RinseWashLiner

FW2:2, 5x

Enabled:	True
Runtime:	1
Autosampler:	PAL:LeftHead
WashLiner:	Fast Wash 2:2
Cycles:	5

9: MoveToHome

LeftHead - Move to Home to mount LS1

Runtime:	70
ScheduledAfter:	-1
Autosampler:	%L_AUTOSAMPLER%
Tool:	LS 1

10: MoveToHome

RightHead - Move to Home to mount required tool holder

Runtime:	2
ScheduledAfter:	-2
Autosampler:	%R_AUTOSAMPLER%
Tool:	%TOOL_1mLR%

11: CleanSyringe

Enabled:	True
Runtime:	1
Autosampler:	PAL:LeftHead
Tool:	%TOOL_1mLL%
WashStation:	Fast Wash 1:1
CleanCycles:	3
FillLevel:	100%

12: CleanSyringe

Enabled:	True
Runtime:	1
Autosampler:	PAL:RightHead
Tool:	%TOOL_1mLR%
WashStation:	Fast Wash 2:1
CleanCycles:	3
FillLevel:	100%

13: Repeat

****OuterLoop_3*** Looping through sets of 8 samples and the last set of either 8 or less samples*

Count:	| IF %NumVials% Mod 8=0 THEN Return %NumVials% / 8 ELSE Return (((%NumVials% - (%NumVials% Mod 8)) / 8)+1) |
Token:	%Outer_Loop3%

14: WriteToLogfile

counter logging OuterLoop_3

Enabled:	| IF %YNCounterLog%=True THEN Return True ELSE Return False |
Runtime:	1
Logfile:	C:\Users\PAL-tower\Documents\Chronos\RunLogs\CounterLogging.txt
Text:	TotalOuterLoop3Number=| IF %NumVials% Mod 8=0 THEN Return %NumVials% / 8 ELSE Return (((%NumVials% - (%NumVials% Mod 8)) / 8)+1) |, CurrentOuterLoop3Number=%Outer_Loop3%
IncludeTimeStamp:	True
Append:	True

15: Repeat

*IL3***InnerLoop*** 1st MTBE Addition & Vortexing - Looping through one set of 8 or less samples*

Count:	| IF %NumVials% Mod 8=0 THEN Return 8 ELSE IF %Outer_Loop3% < (((%NumVials% - (%NumVials% Mod 8)) / 8)+1) THEN Return 8 ELSE Return %NumVials% Mod 8 |
Token:	%Inner_Loop3%

16: WriteToLogfile

counter logging InnerLoop_3

Enabled:	| IF %YNCounterLog%=True THEN Return True ELSE Return False |
Runtime:	1
Logfile:	C:\Users\PAL-tower\Documents\Chronos\RunLogs\CounterLogging.txt
Text:	TotalInnerLoop3Number=| IF %NumVials% Mod 8=0 THEN Return 8 ELSE IF %Outer_Loop3% < (((%NumVials% - (%NumVials% Mod 8)) / 8)+1) THEN Return 8 ELSE Return %NumVials% Mod 8 |, CurrentInnerLoop3Number=%Inner_Loop3%
IncludeTimeStamp:	True
Append:	True

17: SolventModuleSelector

Account for removed MTBE solvent from solvent reservoir

Enabled:	| IF %Inner_Loop3%=1 THEN Return True ELSE Return False |
Runtime:	1
SolventModuleVolume:	100000 µL
MinimumSolventVolume:	%MTBEMinSolvMod% µL
VolumeUsed:	%MTBE1Vol% µL
SolventModuleAction:	UseSolvent
ActionOnAllEmpty:	PauseAndRefill

18: WriteToLogfile

log file entry solvent use from MTBE solvent reservoir

Enabled:	| IF %Inner_Loop3%=1 THEN Return True ELSE Return False |
Runtime:	1
Logfile:	C:\Users\PAL-tower\Documents\Chronos\RunLogs\%FILENAME%
Text:	{jit:-1:ActiveSolventModule}, {jit:-1:SolventRemaining}mL is left
IncludeTimeStamp:	True
Append:	True

19: Transfer

RightHead - transfer "MTBE Add1 Volume" from LargeWash to vial in "SOURCE Tray"

Enabled:	| IF %Inner_Loop3%=1 THEN Return True ELSE Return False |
Runtime:	59
RespectRuntime:	False
Autosampler:	%R_AUTOSAMPLER%
Tool:	%TOOL_1mLR%
Source:	{jit:-2:ActiveSolventModule//Solvent Module1:1}
Destination:	%SRCTRAY%:[%SRCVIAL% + (%Inner_Loop3% - 1) + ((%Outer_Loop3% - 1) * 8)]
Volume:	%MTBE1Vol%
VentDestination:	False
AirVolume:	0 µL
FillVolume:	100 µL
FillSpeed:	%MTBEWash% uL/s
FillStrokes:	%MTBEFillStrokes%
DestinationPenetration:	%VialDispPenDepth%
DestinationEjectSpeed:	%MeH2OWash% uL/s
LeaveDestinationDrawerOpen:	True

20: MoveToHome

RightHead - Move to Home

Enabled:	| IF %Inner_Loop3%=1 THEN Return True ELSE Return False |
Runtime:	8
Autosampler:	%R_AUTOSAMPLER%
Tool:	%TOOL_1mLR%

21: Wait

<just to have a line in loop for ScheduleAfter setting - subsequent processes will start from here>

Runtime:	1

22: Transport

transport from vial tray to Vortexer

Runtime:	1
ScheduledAfter:	-1
Autosampler:	%L_AUTOSAMPLER%
Tool:	%TOOL_1mLL%
Source:	%SRCTRAY%:[%SRCVIAL% + (%Inner_Loop3% - 1) + ((%Outer_Loop3% - 1) * 8)]
Destination:	Item Position 1
LeaveSourceDrawerOpen:	True
LeaveDestinationDrawerOpen:	True

23: ExecuteActivity

Left Head move up so there is no contact

Runtime:	1
ScheduledAfter:	-1
Autosampler:	%L_AUTOSAMPLER%
Tool:	%TOOL_1mLL%
Activity:	MoveRelative
ReferencePoint:	Item Position 1
MovementX:	0 mm
MovementY:	0 mm
MovementZ:	-10 mm
AccelerationFactor:	33 %
DrfOption:	VelAcc
ForceDirectMovement:	False

24: VortexEx

vortexing vial

Runtime:	1
ScheduledAfter:	-1
Autosampler:	%L_AUTOSAMPLER%
MixTime:	%VRTXTIME1%
Speed:	%VRTXSPEED% rpm

25: Wait

RightHead - delay close drawer action in order for vial pickup for vortexing already under way

Enabled:	True
Runtime:	6
ScheduledAfter:	-3

26: ExecuteActivity

RightHead - close open drawer

Enabled:	True
Runtime:	14
ScheduledAfter:	-1
Autosampler:	%R_AUTOSAMPLER%
Tool:	%TOOL_1mLR%
Activity:	CloseOpenDrawers
Stack:	Peltier Stack 1

27: CleanSyringe

Runtime:	1
ScheduledAfter:	-1
Autosampler:	PAL:RightHead
Tool:	%TOOL_1mLR%
WashStation:	Fast Wash 2:1
CleanCycles:	1
FillLevel:	5%

28: MoveToHome

RightHead - Move to Home

Enabled:	True
Runtime:	7
ScheduledAfter:	-1
Autosampler:	%R_AUTOSAMPLER%
Tool:	%TOOL_1mLR%

29: Transport

transport vial back from vortexer to tray

Runtime:	1
Autosampler:	%L_AUTOSAMPLER%
Tool:	%TOOL_1mLL%
Source:	Item Position 1
Destination:	%SRCTRAY%:[%SRCVIAL% + (%Inner_Loop3% - 1) + ((%Outer_Loop3% - 1) * 8)]
LeaveSourceDrawerOpen:	True
LeaveDestinationDrawerOpen:	False

30: MoveToHome

LeftHead - Move to Home

Enabled:	True
Runtime:	7
ScheduledAfter:	-1
Autosampler:	%L_AUTOSAMPLER%
Tool:	%TOOL_1mLL%

31: SolventModuleSelector

Account for removed MTBE solvent from solvent reservoir

Enabled:	| IF %Inner_Loop3% < 8 THEN IF (%SRCVIAL% + (%Inner_Loop3% - 1) + ((%Outer_Loop3% - 1) * 8)) < %NumVials% THEN Return True ELSE Return False ELSE Return
Runtime:	1
RespectRuntime:	False
ScheduledAfter:	-1
SolventModuleVolume:	100000 µL
MinimumSolventVolume:	%MTBEMinSolvMod% µL
VolumeUsed:	%MTBE1Vol% µL
SolventModuleAction:	UseSolvent
ActionOnAllEmpty:	PauseAndRefill

32: WriteToLogfile

log file entry solvent use from MTBE solvent reservoir

Enabled:	| IF %Inner_Loop3% < 8 THEN IF (%SRCVIAL% + (%Inner_Loop3% - 1) + ((%Outer_Loop3% - 1) * 8)) < %NumVials% THEN Return True ELSE Return False ELSE Return
Runtime:	1
ScheduledAfter:	-1
Logfile:	C:\Users\PAL-tower\Documents\Chronos\RunLogs\%FILENAME%
Text:	{jit:-1:ActiveSolventModule}, {jit:-1:SolventRemaining}mL is left
IncludeTimeStamp:	True
Append:	True

33: Transfer

RightHead New - transfer "MTBE Add1 Volume" from LargeWash to vial in "SOURCE Tray"

Enabled:	| IF %Inner_Loop3% < 8 THEN IF (%SRCVIAL% + (%Inner_Loop3% - 1) + ((%Outer_Loop3% - 1) * 8)) < (%SRCVIAL% -1 + %NumVials%) THEN Return True ELSE Return False ELSE Return False |
Runtime:	46
RespectRuntime:	False
ScheduledAfter:	-1
Autosampler:	%R_AUTOSAMPLER%
Tool:	%TOOL_1mLR%
Source:	{jit:-2:ActiveSolventModule//Solvent Module1:1}
Destination:	%SRCTRAY%:[%SRCVIAL% + (%Inner_Loop3% - 1) + ((%Outer_Loop3% - 1) * 8)+1]
Volume:	%MTBE1Vol%
VentDestination:	False
AirVolume:	0 µL
FillVolume:	100 µL
FillSpeed:	%MTBEWash% uL/s
FillStrokes:	%MTBEFillStrokes%
DestinationPenetration:	%VialDispPenDepth%
DestinationEjectSpeed:	%MeH2OWash% uL/s
LeaveDestinationDrawerOpen:	True

34: MoveToHome

RightHead - Move to Home

Enabled:	| IF %Inner_Loop3% < 8 THEN IF (%SRCVIAL% + (%Inner_Loop3% - 1) + ((%Outer_Loop3% - 1) * 8)) < (%SRCVIAL% -1 + %NumVials%) THEN Return True ELSE Return False ELSE Return False |
Runtime:	8
ScheduledAfter:	-1
Autosampler:	%R_AUTOSAMPLER%
Tool:	%TOOL_1mLR%

35: RepeatEnd

*IL3***InnerLoop*** 1st MTBE Addition & Vortexing - Looping through one set of 8 or less samples*

36: MoveToHome

LeftHead - Move to Home

Runtime:	1
Autosampler:	%L_AUTOSAMPLER%
Tool:	%TOOL_1mLL%

37: Repeat

*IL4***InnerLoop*** Transport Vials into Centrifuge - Looping through one set of 8 or less samples*

Count:	| IF %NumVials% Mod 8=0 THEN Return 8 ELSE IF %Outer_Loop3% < (((%NumVials% - (%NumVials% Mod 8)) / 8)+1) THEN Return 8 ELSE Return %NumVials% Mod 8 |
Token:	%Inner_Loop4%

38: WriteToLogfile

counter logging InnerLoop_4

Enabled:	| IF %YNCounterLog%=True THEN Return True ELSE Return False |
Runtime:	1
Logfile:	C:\Users\PAL-tower\Documents\Chronos\RunLogs\CounterLogging.txt
Text:	TotalInnerLoop4Number=| IF %NumVials% Mod 8=0 THEN Return 8 ELSE IF %Outer_Loop3% < (((%NumVials% - (%NumVials% Mod 8)) / 8)+1) THEN Return 8 ELSE Return %NumVials% Mod 8 |, CurrentInnerLoop4Number=%Inner_Loop4%
IncludeTimeStamp:	True
Append:	True

39: Transport

RightHead - Transport Vial into Centrifuge

Runtime:	28
Autosampler:	%R_AUTOSAMPLER%
Tool:	%TOOL_1mLR%
Source:	%SRCTRAY%:[%SRCVIAL% + (%Inner_Loop4% - 1) + ((%Outer_Loop3% - 1) * 8)]
Destination:	Centrifuge 1:%Inner_Loop4%

40: RepeatEnd

*IL4***InnerLoop*** Transport Vials into Centrifuge - Looping through one set of 8 or less samples*

41: MoveToHome

RightHead - Move to Home

Enabled:	True
Runtime:	7
Autosampler:	%R_AUTOSAMPLER%
Tool:	%TOOL_1mLR%

42: Wait

LeftHead - delay close drawer action in order for vial pickup for vortexing already under way

Runtime:	1
ScheduledAfter:	-1

43: ExecuteActivity

LeftHead - close open drawer

Runtime:	14
ScheduledAfter:	-1
Autosampler:	%L_AUTOSAMPLER%
Tool:	%TOOL_1mLL%
Activity:	CloseOpenDrawers
Stack:	Peltier Stack 1

44: MoveToHome

LeftHead - Move to Home

Runtime:	10
ScheduledAfter:	-1
Autosampler:	%L_AUTOSAMPLER%
Tool:	%TOOL_1mLL%

45: ExecuteActivity

Start Centrifuge

Runtime:	9
ScheduledAfter:	-3
Autosampler:	%R_AUTOSAMPLER%
Tool:	%TOOL_1mLR%
Activity:	SetCentrifuge
Target:	Centrifuge 1
Speed:	%CENTSPEED%
gForce:	0
State:	On
WaitForConstSpeed:	False

46: Wait

Centrifugation time

Runtime:	[%CENTTIME%*60]
ScheduledAfter:	-1

47: ExecuteActivity

Stop Centrifuge

Runtime:	23
ScheduledAfter:	-1
Autosampler:	%R_AUTOSAMPLER%
Tool:	%TOOL_1mLR%
Activity:	SetCentrifuge
Target:	Centrifuge 1
Speed:	
gForce:	0
State:	Off
WaitForConstSpeed:	False

48: Repeat

*IL5***InnerLoop*** Transport Vials from Centrifuge - Looping through one set of 8 or less samples*

Count:	| IF %NumVials% Mod 8=0 THEN Return 8 ELSE IF %Outer_Loop3% < (((%NumVials% - (%NumVials% Mod 8)) / 8)+1) THEN Return 8 ELSE Return %NumVials% Mod 8 |
Token:	%Inner_Loop5%

49: WriteToLogfile

counter logging InnerLoop_5

Enabled:	| IF %YNCounterLog%=True THEN Return True ELSE Return False |
Runtime:	1
Logfile:	C:\Users\PAL-tower\Documents\Chronos\RunLogs\CounterLogging.txt
Text:	TotalInnerLoop5Number=| IF %NumVials% Mod 8=0 THEN Return 8 ELSE IF %Outer_Loop3% < (((%NumVials% - (%NumVials% Mod 8)) / 8)+1) THEN Return 8 ELSE Return %NumVials% Mod 8 |, CurrentInnerLoop5Number=%Inner_Loop5%
IncludeTimeStamp:	True
Append:	True

50: Transport

RightHead - Transport Vial from Centrifuge

Runtime:	36
Autosampler:	%R_AUTOSAMPLER%
Tool:	%TOOL_1mLR%
Source:	Centrifuge 1:%Inner_Loop5%
Destination:	%SRCTRAY%:[%SRCVIAL% + (%Inner_Loop5% - 1) + ((%Outer_Loop3% - 1) * 8)]
LeaveSourceDrawerOpen:	True
LeaveDestinationDrawerOpen:	True

51: RepeatEnd

*IL5***InnerLoop*** Transport Vials from Centrifuge - Looping through one set of 8 or less samples*

52: ExecuteActivity

RightHead - close open drawer

Runtime:	9
Autosampler:	%R_AUTOSAMPLER%
Tool:	%TOOL_1mLR%
Activity:	CloseOpenDrawers
Stack:	Peltier Stack 1

53: MoveToHome

RightHead - Move to Home

Enabled:	True
Runtime:	9
Autosampler:	%R_AUTOSAMPLER%
Tool:	%TOOL_1mLR%

54: Repeat

*IL6***InnerLoop*** 1st Sampling & 2nd MTBE Addition - Looping through one set of 8 or less samples*

Count:	| IF %NumVials% Mod 8=0 THEN Return 8 ELSE IF %Outer_Loop3% < (((%NumVials% - (%NumVials% Mod 8)) / 8)+1) THEN Return 8 ELSE Return %NumVials% Mod 8 |
Token:	%Inner_Loop6%

55: WriteToLogfile

counter logging InnerLoop_6

Enabled:	| IF %YNCounterLog%=True THEN Return True ELSE Return False |
Runtime:	1
Logfile:	C:\Users\PAL-tower\Documents\Chronos\RunLogs\CounterLogging.txt
Text:	TotalInnerLoop6Number=| IF %NumVials% Mod 8=0 THEN Return 8 ELSE IF %Outer_Loop3% < (((%NumVials% - (%NumVials% Mod 8)) / 8)+1) THEN Return 8 ELSE Return %NumVials% Mod 8 |, CurrentInnerLoop6Number=%Inner_Loop6%
IncludeTimeStamp:	True
Append:	True

56: Wait

<just to have a first line in loop without a ScheduleAfter setting - subsequent processes will start from here>

Runtime:	1

57: Transfer

LeftHead - transfer "DestA 1of2 Volume" from "SOURCE Tray" to "DestA Tray"

Enabled:	True
Runtime:	86
ScheduledAfter:	-1
Autosampler:	%L_AUTOSAMPLER%
Tool:	%TOOL_1mLL%
Source:	%SRCTRAY%:[%SRCVIAL% + (%Inner_Loop6% - 1) + ((%Outer_Loop3% - 1) * 8)]
Destination:	%TRAYA%:[%VIALA% + (%Inner_Loop6% - 1) + ((%Outer_Loop3% - 1) * 8)]
Volume:	%DestA1Vol%
VentDestination:	False
AirVolume:	0 µL
SourcePenetration:	%TopAspPenDepth%
FillSpeed:	%FillSpeed1000% uL/s
FillStrokes:	0
DestinationPenetration:	%VialDispPenDepth%
DestinationEjectSpeed:	%MTBEWash% uL/s

58: CleanSyringe

LeftHead - clean syringe in Wash1 as many as "Post Clean With Solvent 1" times

Runtime:	42
ScheduledAfter:	-1
Autosampler:	%L_AUTOSAMPLER%
Tool:	%TOOL_1mLL%
WashStation:	Fast Wash 1:1
CleanCycles:	2
FillSpeed:	%MTBEWash% uL/s
EjectSpeed:	%MTBEWash% uL/s

59: CleanSyringe

LeftHead - clean syringe in Wash2 as many as "Post Clean With Solvent 2" times

Enabled:	False
Runtime:	%VRTXWSHDEL%
RespectRuntime:	True
ScheduledAfter:	-1
Autosampler:	%L_AUTOSAMPLER%
Tool:	%TOOL_1mLL%
WashStation:	Fast Wash 1:2
CleanCycles:	%PostCleanSolvent2%
FillSpeed:	%MTBEWash% uL/s

60: Wait

RightHead - delay MTBE addition until after transfer

Enabled:	True
Runtime:	10
ScheduledAfter:	-3

61: SolventModuleSelector

Account for removed MTBE solvent from solvent reservoir

Enabled:	True
Runtime:	1
ScheduledAfter:	-1
SolventModuleVolume:	100000 µL
MinimumSolventVolume:	%MTBEMinSolvMod% µL
VolumeUsed:	%MTBE2Vol% µL
SolventModuleAction:	UseSolvent
ActionOnAllEmpty:	PauseAndRefill

62: WriteToLogfile

log file entry solvent use from MTBE solvent reservoir

Enabled:	True
Runtime:	1
ScheduledAfter:	-1
Logfile:	C:\Users\PAL-tower\Documents\Chronos\RunLogs\%FILENAME%
Text:	{jit:-1:ActiveSolventModule}, {jit:-1:SolventRemaining}mL is left
IncludeTimeStamp:	True
Append:	True

63: Transfer

RightHead - transfer "MTBE Add2 Volume" from LargeWash to vial in "SOURCE Tray"

Enabled:	True
Runtime:	141
RespectRuntime:	False
ScheduledAfter:	-1
Autosampler:	%R_AUTOSAMPLER%
Tool:	%TOOL_1mLR%
Source:	{jit:-2:ActiveSolventModule//Solvent Module1:1}
Destination:	%SRCTRAY%:[%SRCVIAL% + (%Inner_Loop6% - 1) + ((%Outer_Loop3% - 1) * 8)]
Volume:	%MTBE2Vol%
VentDestination:	False
FillVolume:	200 µL
FillSpeed:	%MTBEWash% uL/s
FillStrokes:	%MTBEFillStrokes%
DestinationPenetration:	%VialDispPenDepth%
DestinationEjectSpeed:	%MeH2OWash% uL/s

64: CleanSyringe

Runtime:	1
ScheduledAfter:	-1
Autosampler:	PAL:RightHead
Tool:	%TOOL_1mLR%
WashStation:	Fast Wash 2:1
CleanCycles:	1
FillLevel:	5%

65: MoveToHome

RightHead - Move to Home

Enabled:	True
Runtime:	8
ScheduledAfter:	-1
Autosampler:	%R_AUTOSAMPLER%
Tool:	%TOOL_1mLR%

66: RepeatEnd

*IL6***InnerLoop*** 1st Sampling & 2nd MTBE Addition - Looping through one set of 8 or less samples*

67: Repeat

*IL7***InnerLoop*** Vortexing - Looping through one set of 8 or less samples*

Count:	| IF %NumVials% Mod 8=0 THEN Return 8 ELSE IF %Outer_Loop3% < (((%NumVials% - (%NumVials% Mod 8)) / 8)+1) THEN Return 8 ELSE Return %NumVials% Mod 8 |
Token:	%Inner_Loop7%

68: WriteToLogfile

counter logging InnerLoop_7

Enabled:	| IF %YNCounterLog%=True THEN Return True ELSE Return False |
Runtime:	1
Logfile:	C:\Users\PAL-tower\Documents\Chronos\RunLogs\CounterLogging.txt
Text:	TotalInnerLoop7Number=| IF %NumVials% Mod 8=0 THEN Return 8 ELSE IF %Outer_Loop3% < (((%NumVials% - (%NumVials% Mod 8)) / 8)+1) THEN Return 8 ELSE Return %NumVials% Mod 8 |, CurrentInnerLoop7Number=%Inner_Loop7%
IncludeTimeStamp:	True
Append:	True

69: Wait

<just to have a first line in loop without a ScheduleAfter setting - subsequent processes will start from here>

Runtime:	1

70: Transport

transport from vial tray to Vortexer

Runtime:	1
ScheduledAfter:	-1
Autosampler:	%L_AUTOSAMPLER%
Tool:	%TOOL_1mLL%
Source:	%SRCTRAY%:[%SRCVIAL% + (%Inner_Loop7% - 1) + ((%Outer_Loop3% - 1) * 8)]
Destination:	Item Position 1
LeaveSourceDrawerOpen:	True
LeaveDestinationDrawerOpen:	True

71: ExecuteActivity

Left Head move up so there is no contact

Runtime:	1
ScheduledAfter:	-1
Autosampler:	%L_AUTOSAMPLER%
Tool:	%TOOL_1mLL%
Activity:	MoveRelative
ReferencePoint:	Item Position 1
MovementX:	0 mm
MovementY:	0 mm
MovementZ:	-10 mm
AccelerationFactor:	33 %
DrfOption:	VelAcc
ForceDirectMovement:	False

72: VortexEx

vortexing vial

Runtime:	1
ScheduledAfter:	-1
Autosampler:	%L_AUTOSAMPLER%
MixTime:	%VRTXTIME2%
Speed:	%VRTXSPEED% rpm

73: Wait

RightHead - delay close drawer action in order for vial pickup for vortexing already under way

Runtime:	10
ScheduledAfter:	-3

74: ExecuteActivity

RightHead - close open drawer

Runtime:	19
ScheduledAfter:	-1
Autosampler:	%R_AUTOSAMPLER%
Tool:	%TOOL_1mLR%
Activity:	CloseOpenDrawers
Stack:	Peltier Stack 1

75: MoveToHome

RightHead - move to home position

Runtime:	10
ScheduledAfter:	-1
Autosampler:	%R_AUTOSAMPLER%
Tool:	%TOOL_1mLR%

76: Transport

transport vial back from vortexer to tray

Runtime:	1
ScheduledAfter:	-4
Autosampler:	%L_AUTOSAMPLER%
Tool:	%TOOL_1mLL%
Source:	Item Position 1
Destination:	%SRCTRAY%:[%SRCVIAL% + (%Inner_Loop7% - 1) + ((%Outer_Loop3% - 1) * 8)]
LeaveSourceDrawerOpen:	True
LeaveDestinationDrawerOpen:	True

77: RepeatEnd

*IL7***InnerLoop*** Vortexing - Looping through one set of 8 or less samples*

78: MoveToHome

LeftHead - Move to Home

Runtime:	7
Autosampler:	%L_AUTOSAMPLER%
Tool:	%TOOL_1mLL%

79: Repeat

*IL8***InnerLoop*** Transport Vials into Centrifuge - Looping through one set of 8 or less samples*

Count:	| IF %NumVials% Mod 8=0 THEN Return 8 ELSE IF %Outer_Loop3% < (((%NumVials% - (%NumVials% Mod 8)) / 8)+1) THEN Return 8 ELSE Return %NumVials% Mod 8 |
Token:	%Inner_Loop8%

80: WriteToLogfile

counter logging InnerLoop_8

Enabled:	| IF %YNCounterLog%=True THEN Return True ELSE Return False |
Runtime:	1
Logfile:	C:\Users\PAL-tower\Documents\Chronos\RunLogs\CounterLogging.txt
Text:	TotalInnerLoop8Number=| IF %NumVials% Mod 8=0 THEN Return 8 ELSE IF %Outer_Loop3% < (((%NumVials% - (%NumVials% Mod 8)) / 8)+1) THEN Return 8 ELSE Return %NumVials% Mod 8 |, CurrentInnerLoop8Number=%Inner_Loop8%
IncludeTimeStamp:	True
Append:	True

81: Transport

RightHead - Transport Vial into Centrifuge

Runtime:	24
Autosampler:	%R_AUTOSAMPLER%
Tool:	%TOOL_1mLR%
Source:	%SRCTRAY%:[%SRCVIAL% + (%Inner_Loop8% - 1) + ((%Outer_Loop3% - 1) * 8)]
Destination:	Centrifuge 1:%Inner_Loop8%

82: RepeatEnd

*IL8***InnerLoop*** Transport Vials into Centrifuge - Looping through one set of 8 or less samples*

83: MoveToHome

RightHead - Move to Home

Enabled:	True
Runtime:	9
Autosampler:	%R_AUTOSAMPLER%
Tool:	%TOOL_1mLR%

84: Wait

LeftHead - delay close drawer action in order for vial pickup for vortexing already under way

Runtime:	1
ScheduledAfter:	-1

85: ExecuteActivity

LeftHead - close open drawer

Runtime:	14
ScheduledAfter:	-1
Autosampler:	%L_AUTOSAMPLER%
Tool:	%TOOL_1mLL%
Activity:	CloseOpenDrawers
Stack:	Peltier Stack 1

86: MoveToHome

LeftHead - Move to Home

Runtime:	9
ScheduledAfter:	-1
Autosampler:	%L_AUTOSAMPLER%
Tool:	%TOOL_1mLL%

87: ExecuteActivity

Start Centrifuge

Runtime:	10
ScheduledAfter:	-3
Autosampler:	%R_AUTOSAMPLER%
Tool:	%TOOL_1mLR%
Activity:	SetCentrifuge
Target:	Centrifuge 1
Speed:	%CENTSPEED%
gForce:	0
State:	On
WaitForConstSpeed:	False

88: Wait

Centrifugation time

Runtime:	[%CENTTIME%*60]
ScheduledAfter:	-1

89: ExecuteActivity

Stop Centrifuge

Runtime:	10
ScheduledAfter:	-1
Autosampler:	%R_AUTOSAMPLER%
Tool:	%TOOL_1mLR%
Activity:	SetCentrifuge
Target:	Centrifuge 1
Speed:	
gForce:	0
State:	Off
WaitForConstSpeed:	False

90: Repeat

*IL9***InnerLoop*** Transport Vials from Centrifuge - Looping through one set of 8 or less samples*

Count:	| IF %NumVials% Mod 8=0 THEN Return 8 ELSE IF %Outer_Loop3% < (((%NumVials% - (%NumVials% Mod 8)) / 8)+1) THEN Return 8 ELSE Return %NumVials% Mod 8 |
Token:	%Inner_Loop9%

91: WriteToLogfile

counter logging InnerLoop_9

Enabled:	| IF %YNCounterLog%=True THEN Return True ELSE Return False |
Runtime:	1
Logfile:	C:\Users\PAL-tower\Documents\Chronos\RunLogs\CounterLogging.txt
Text:	TotalInnerLoop9Number=| IF %NumVials% Mod 8=0 THEN Return 8 ELSE IF %Outer_Loop3% < (((%NumVials% - (%NumVials% Mod 8)) / 8)+1) THEN Return 8 ELSE Return %NumVials% Mod 8 |, CurrentInnerLoop9Number=%Inner_Loop9%
IncludeTimeStamp:	True
Append:	True

92: Transport

RightHead - Transport Vial from Centrifuge

Runtime:	37
Autosampler:	%R_AUTOSAMPLER%
Tool:	%TOOL_1mLR%
Source:	Centrifuge 1:%Inner_Loop9%
Destination:	%SRCTRAY%:[%SRCVIAL% + (%Inner_Loop9% - 1) + ((%Outer_Loop3% - 1) * 8)]
LeaveSourceDrawerOpen:	True
LeaveDestinationDrawerOpen:	True

93: RepeatEnd

*IL9***InnerLoop*** Transport Vials from Centrifuge - Looping through one set of 8 or less samples*

94: ExecuteActivity

RightHead - close open drawer

Runtime:	9
Autosampler:	%R_AUTOSAMPLER%
Tool:	%TOOL_1mLR%
Activity:	CloseOpenDrawers
Stack:	Peltier Stack 1

95: MoveToHome

RightHead - Move to Home

Enabled:	True
Runtime:	9
Autosampler:	%R_AUTOSAMPLER%
Tool:	%TOOL_1mLR%

96: Repeat

*IL10***InnerLoop*** 2nd Sampling - Looping through one set of 8 or less samples*

Count:	| IF %NumVials% Mod 8=0 THEN Return 8 ELSE IF %Outer_Loop3% < (((%NumVials% - (%NumVials% Mod 8)) / 8)+1) THEN Return 8 ELSE Return %NumVials% Mod 8 |
Token:	%Inner_Loop10%

97: WriteToLogfile

counter logging InnerLoop_10

Enabled:	| IF %YNCounterLog%=True THEN Return True ELSE Return False |
Runtime:	1
Logfile:	C:\Users\PAL-tower\Documents\Chronos\RunLogs\CounterLogging.txt
Text:	TotalInnerLoop10Number=| IF %NumVials% Mod 8=0 THEN Return 8 ELSE IF %Outer_Loop3% < (((%NumVials% - (%NumVials% Mod 8)) / 8)+1) THEN Return 8 ELSE Return %NumVials% Mod 8 |, CurrentInnerLoop10Number=%Inner_Loop10%
IncludeTimeStamp:	True
Append:	True

98: Transfer

LeftHead - transfer "DestA 2of2 Volume" from "SOURCE Tray" to "DestA Tray"

Enabled:	| IF %Inner_Loop10%=1 THEN Return True ELSE Return False |
Runtime:	66
RespectRuntime:	False
Autosampler:	%L_AUTOSAMPLER%
Tool:	%TOOL_1mLL%
Source:	%SRCTRAY%:[%SRCVIAL% + (%Inner_Loop10% - 1) + ((%Outer_Loop3% - 1) * 8)]
Destination:	%TRAYA%:[%VIALA% + (%Inner_Loop10% - 1) + ((%Outer_Loop3% - 1) * 8)]
Volume:	%DestA2Vol%
VentDestination:	False
DestinationZRetractValue:	0 mm
AirVolume:	0 µL
SourcePenetration:	%TopAspPenDepth2%
FillSpeed:	%FillSpeed1000%
FillStrokes:	%SmplFillStrokes%
DestinationPenetration:	%VialDispPenDepth%
DestinationEjectSpeed:	%MTBEWash% uL/s

99: Wait

placeholder for right head

Runtime:	1
ScheduledAfter:	-1

100: CleanSyringe

LeftHead - clean syringe in Wash1 as many as "Post Clean With Solvent 1" times

Enabled:	| IF %Inner_Loop10%=1 THEN Return True ELSE Return False |
Runtime:	32
ScheduledAfter:	-2
Autosampler:	%L_AUTOSAMPLER%
Tool:	%TOOL_1mLL%
WashStation:	Fast Wash 1:1
CleanCycles:	2
FillLevel:	100%
FillSpeed:	%MTBEWash% uL/s
EjectSpeed:	100 uL/s

101: Transfer

RightHead - transfer "DestB Volume" from "SOURCE Tray" to "DestB Tray"

Enabled:	True
Runtime:	81
ScheduledAfter:	-2
Autosampler:	%R_AUTOSAMPLER%
Tool:	%TOOL_1mLR%
Source:	%SRCTRAY%:[%SRCVIAL% + (%Inner_Loop10% - 1) + ((%Outer_Loop3% - 1) * 8)]
Destination:	%TRAYB%:[%VIALB% + (%Inner_Loop10% - 1) + ((%Outer_Loop3% - 1) * 8)]
Volume:	%DestBVol%
VentDestination:	False
SourcePenetration:	%BotAspPenDepth%
FillSpeed:	%FillSpeed1000%
FillStrokes:	%SmplFillStrokes%
DestinationPenetration:	%VialDispPenDepth%
DestinationEjectSpeed:	%MTBEWash% uL/s

102: CleanSyringe

RightHead - clean syringe in Wash2 as many as "Post Clean With Solvent 2" times

Runtime:	26
ScheduledAfter:	-1
Autosampler:	%R_AUTOSAMPLER%
Tool:	%TOOL_1mLR%
WashStation:	Fast Wash 2:2
CleanCycles:	2
FillLevel:	100%
FillSpeed:	%MeH2OWash% uL/s
EjectSpeed:	100 uL/s

103: Transfer

LeftHead - transfer "DestA 2of2 Volume" from "SOURCE Tray" to "DestA Tray"

Enabled: | IF %Inner_Loop10% < 8 THEN IF (%SRCVIAL% + (%Inner_Loop10% - 1) + ((%Outer_Loop3% - 1) * 8)) < (%SRCVIAL% + %NumVials% -1) THEN Return True ELSE Return False ELSE Return False |

Runtime: 66

RespectRuntime: False

ScheduledAfter: -2

Autosampler: %L_AUTOSAMPLER%

Tool: %TOOL_1mLL%

Source: %SRCTRAY%:[%SRCVIAL% + (%Inner_Loop10% - 1) + ((%Outer_Loop3% - 1) * 8)+1]

Destination: %TRAYA%:[%VIALA% + (%Inner_Loop10% - 1) + ((%Outer_Loop3% - 1) * 8)+1]

Volume: %DestA2Vol%

VentDestination: False

DestinationZRetractValue: 0 mm

AirVolume: 0 µL

SourcePenetration: %TopAspPenDepth2%

FillSpeed: 10 uL/s

FillStrokes: %SmplFillStrokes%

DestinationPenetration: %VialDispPenDepth%

DestinationEjectSpeed: %MTBEWash% uL/s

104: CleanSyringe

LeftHead - clean syringe in Wash1 as many as "Post Clean With Solvent 1" times

Enabled: | IF %Inner_Loop10% < 8 THEN IF (%SRCVIAL% + (%Inner_Loop10% - 1) + ((%Outer_Loop3% - 1) * 8)) < (%SRCVIAL% + %NumVials% -1) THEN Return True ELSE Return False ELSE Return False |

Runtime: 32

ScheduledAfter: -1

Autosampler: %L_AUTOSAMPLER%

Tool: %TOOL_1mLL%

WashStation: Fast Wash 1:1

CleanCycles: 2

FillLevel: 100%

FillSpeed: %MTBEWash% uL/s

EjectSpeed: 100 uL/s

105: RepeatEnd

*IL10***InnerLoop*** 2nd Sampling - Looping through one set of 8 or less samples*

106: MoveToHome

LeftHead - Move to Home

Runtime:	6
Autosampler:	%L_AUTOSAMPLER%
Tool:	%TOOL_1mLL%

107: RepeatEnd

****OuterLoop_3*** Looping through sets of 8 samples and the last set of either 8 or less samples*

108: CleanSyringe

Enabled:	True
Runtime:	1
Autosampler:	PAL:LeftHead
Tool:	%TOOL_1mLL%
WashStation:	Fast Wash 1:1
CleanCycles:	3
FillLevel:	100%

109: CleanSyringe

Enabled:	True
Runtime:	1
Autosampler:	PAL:LeftHead
Tool:	%TOOL_1mLL%
WashStation:	Fast Wash 1:2
CleanCycles:	3
FillLevel:	100%

110: CleanSyringe

Enabled:	True
Runtime:	1
Autosampler:	PAL:RightHead
Tool:	%TOOL_1mLR%
WashStation:	Fast Wash 2:1
CleanCycles:	3
FillLevel:	100%

111: CleanSyringe

Enabled:	True
Runtime:	1
Autosampler:	PAL:RightHead
Tool:	%TOOL_1mLR%
WashStation:	Fast Wash 2:2
CleanCycles:	3
FillLevel:	100%

112: ExecuteActivity

Park Left Head Tool

Runtime:	1
ScheduledAfter:	-1
Autosampler:	PAL:LeftHead
Tool:	LS 1
Activity:	ParkTool
Slot:	none
Safe:	False
Shutdown:	False
ReleaseNdlGuideAdapter:	True

113: ExecuteActivity

Park Right Head Tool

Runtime:	1
ScheduledAfter:	-1
Autosampler:	PAL:RightHead
Tool:	LS 2
Activity:	ParkTool
Slot:	none
Safe:	False
Shutdown:	False
ReleaseNdlGuideAdapter:	True
