# Supplementary material for: Automated preparation of plasma lipids, metabolites, and proteins for LC/MS-based analysis of a high-fat diet in mice
Source: J Lipid Res. 2024 Jul 25;65(9):100607. doi: 10.1016/j.jlr.2024.100607 (PMC11399584; doi:10.1016/j.jlr.2024.100607)
Supplement: PAL-ROBOT-ENCLOSURE-ASM_v2_RevC (2).PDF [file mmc12.pdf]

ITEM NO.	PART NUMBER	DESCRIPTION	
1	TABLE-FRAME_V2		1
2	LC1030SS-CB-PHN	ATLAS CASTER	6
3	BOTTOM-POLYMETAL_V2	POLYMETAL	1
4	TABLE-WORK-SURFACE_V2	O.5" TRESPA OR PHENOLIC RESIN	1
5	NARROW-SHELF_V2	POLYMETAL	1
6	COMPUTER-SURFACE_V2	POLYMETAL	1
7	CHILLER-DRAWER_V2		1
8	TOP-PANEL_V2	ACRYLIC (MEDIUM-HIGH IMPACT)	2
9	BI-FOLD-DOOR_V2		8
10	SIDE-PANEL_V2	ACRYLIC (MEDIUM-HIGH IMPACT)	4
11	40_2884	80/20 INC. HANDLE	4
12	CHILLER-DRAWER-SHELF_V2	POLYMETAL	1
13	6603A430	MCMASTER DRAWER SLIDE	1
14	5124K9	MCMASTER PVC DUCT FLANGE	2
18	GH34-0SS	SUGASTUNE ACRYLIC DOOR HINGE	12
19	PAL-ROBOT-ROUGH_V1		1

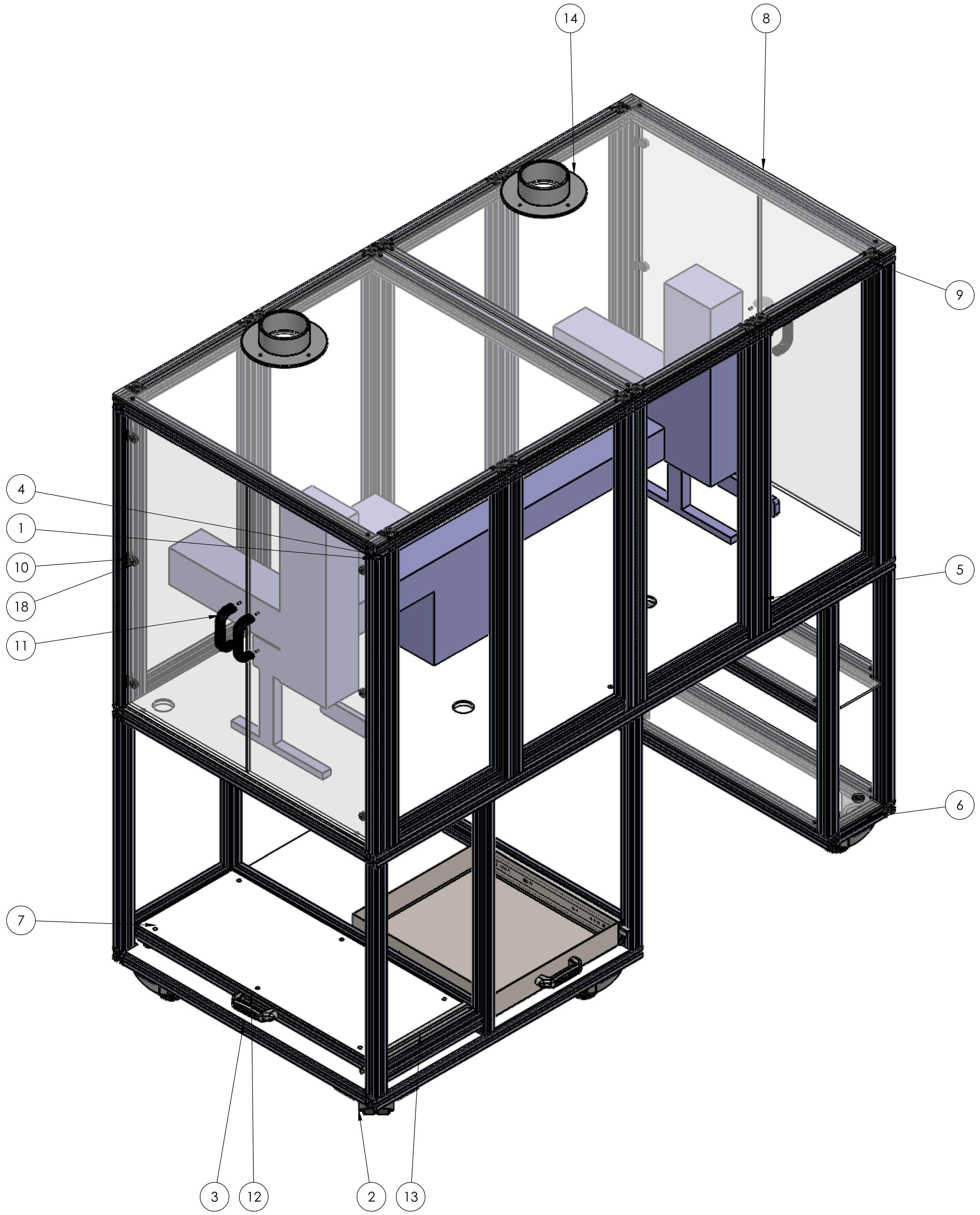

UNLESS OTHERWISE SPECIFIED:
DIMENSIONS ARE IN INCHES
TOLERANCES:
FRACTIONAL ±
ANGULAR: MACH ± BEND ±
TWO PLACE DECIMAL ±
THREE PLACE DECIMAL ±
INTERPRET GEOMETRIC TOLERANCING PER:
MATERIAL
FINISH
NEXT ASSY | USED ON
APPLICATION
DO NOT SCALE DRAWING

	NAME	DATE
DRAWN		
CHECKED		
ENG APPR.		
MFG APPR.		
Q.A.		

COMMENTS:

TITLE:

SIZE | DWG. NO. | REV
PAL-ROBOT-ENCLOSURE-ASM_v2
SCALE: 1:20 WEIGHT: SHEET 1 OF 9

NOTE:
1. These PARTS are NORMAL 40 series (not UL ultra light).

ITEM NO.	PART NUMBER	DESCRIPTION	QTY.
1	TABLE-FRAME_v2		1
		40-4040-UL	12
		40-4080-UL	1
		40-4040-UL	10
		40-4040-UL	4
		40-4040-UL	2
		40-4040-UL	2
		40-4040-UL	2
		40-4040-UL	2
		40-4040-UL	4

ITEM NO.	QTY.	DESCRIPTION	LENGTH
1	12	40-4040-UL	860
2	10	40-4040-UL	710
3	4	40-4040-UL	1850
4	2	40-4040-UL	950
5	2	40-4040-UL	160
6	2	40-4040-UL	940
7	2	40-4040-UL	341.9
8	1	40-4080-UL	860
9	4	40-4040-UL	910

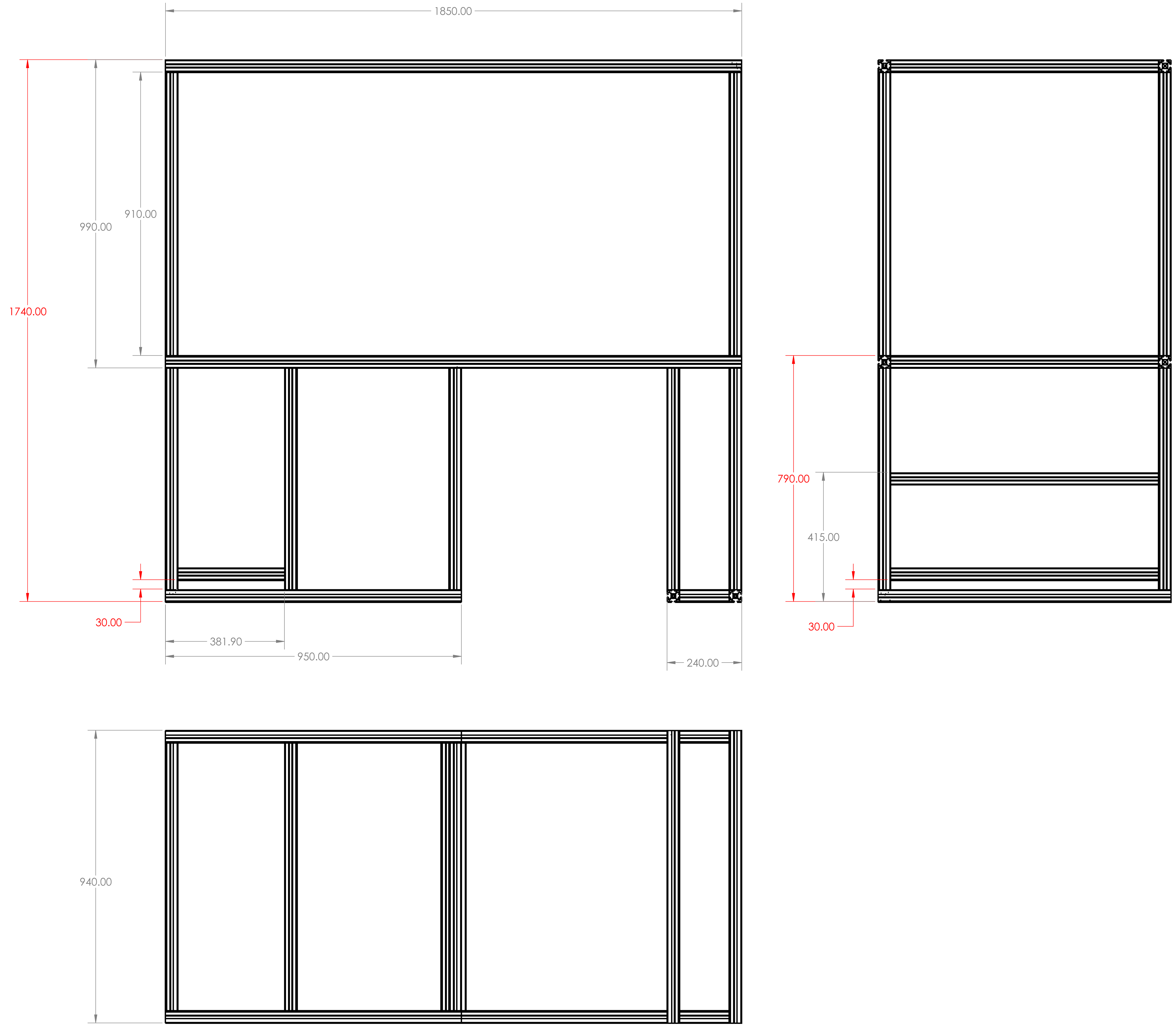

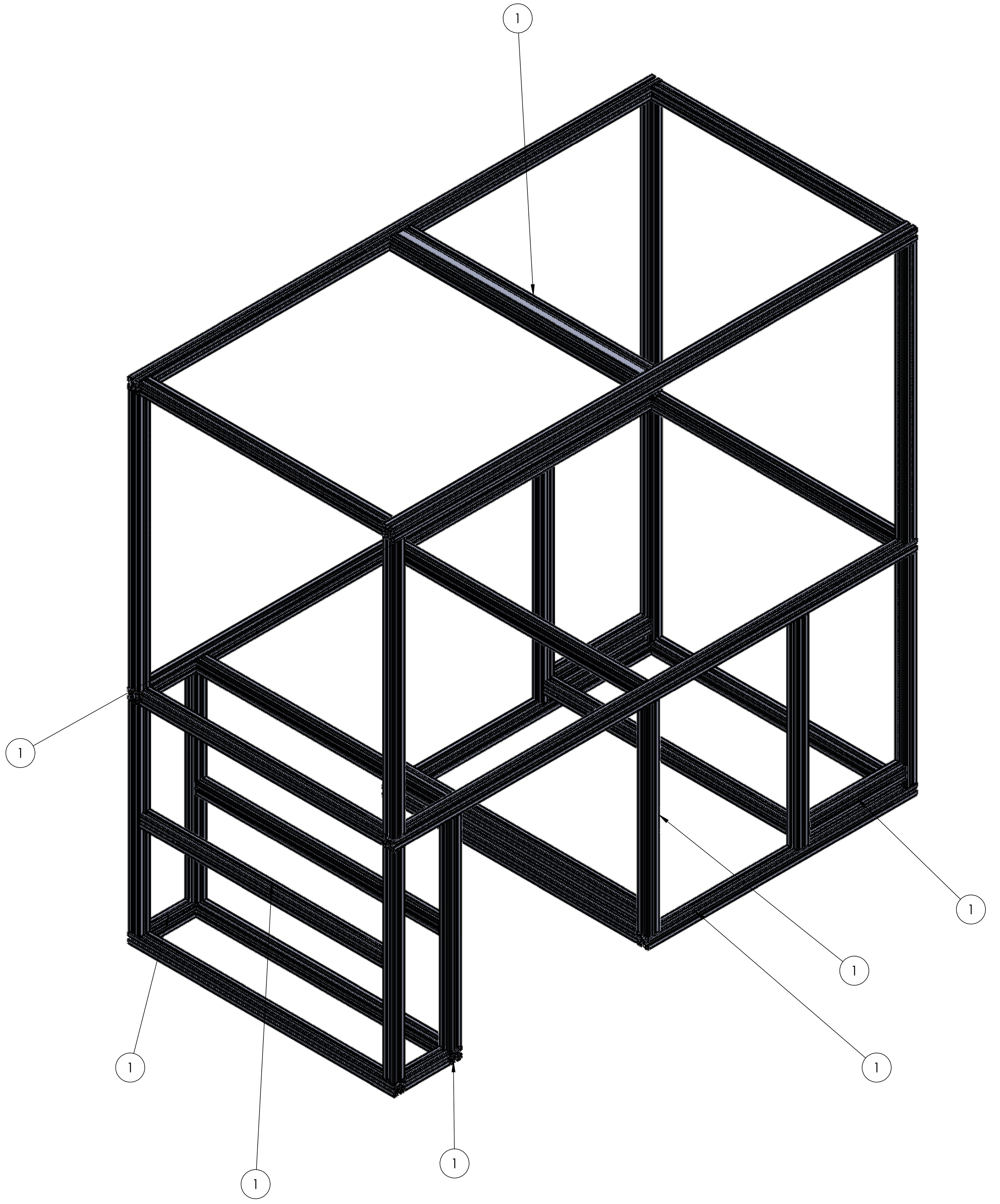

		UNLESS OTHERWISE SPECIFIED:		NAME	DATE	
		DIMENSIONS ARE IN INCHES TOLERANCES: FRACTIONAL ± ANGULAR: MACH ± BEND ± TWO PLACE DECIMAL ± THREE PLACE DECIMAL ±	DRAWN			TITLE:
			CHECKED			
			ENG APPR.			
			MFG APPR.			
		INTERPRET GEOMETRIC TOLERANCING PER:	Q.A.			
		MATERIAL	COMMENTS:			SIZE DWG. NO. REV
NEXT ASSY	USED ON	FINISH				PAL-ROBOT-ENCLOSURE-ASM_v2
APPLICATION		DO NOT SCALE DRAWING				SCALE: 1:20 WEIGHT: SHEET 2 OF 9

NOTES:
1. 1/2" TRESPA
2. 1/8" BEVEL

ITEM NO.	PART NUMBER	DESCRIPTION	QTY.
1	TABLE-WORK-SURFACE_v2	O.5" TRESPA OR PHENOLIC RESIN	1

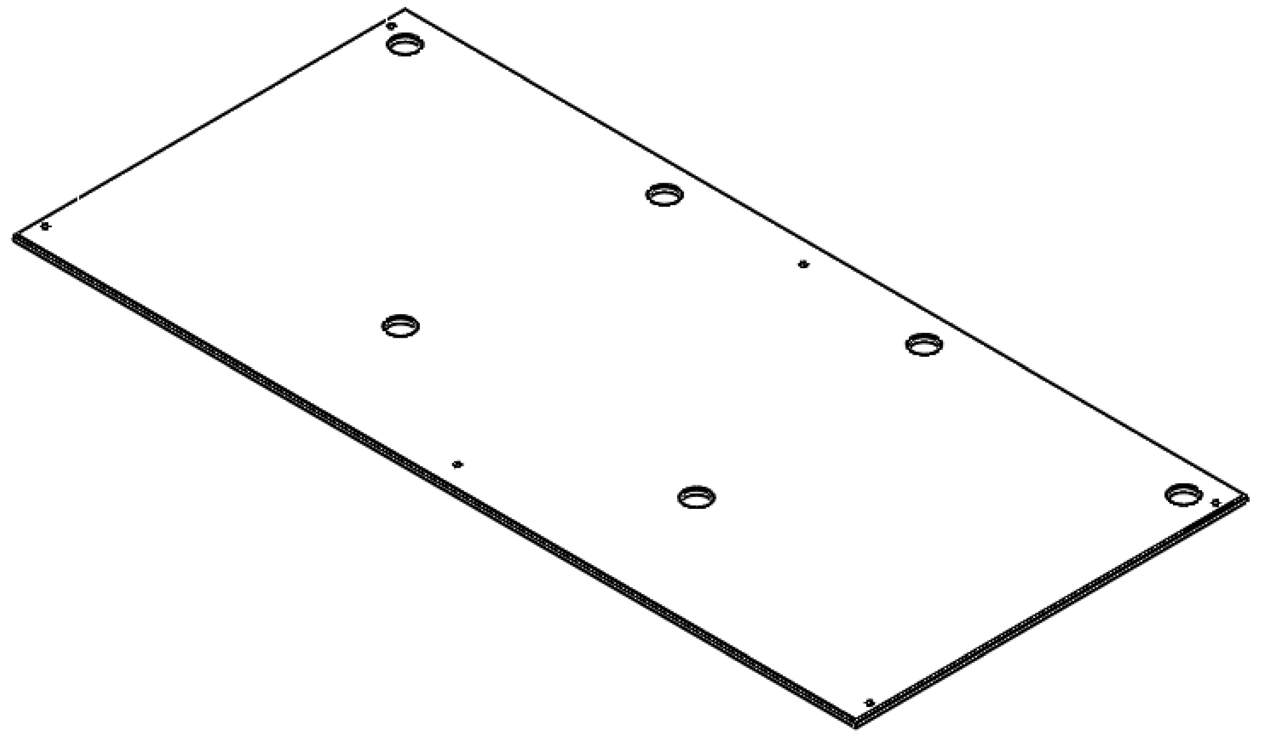

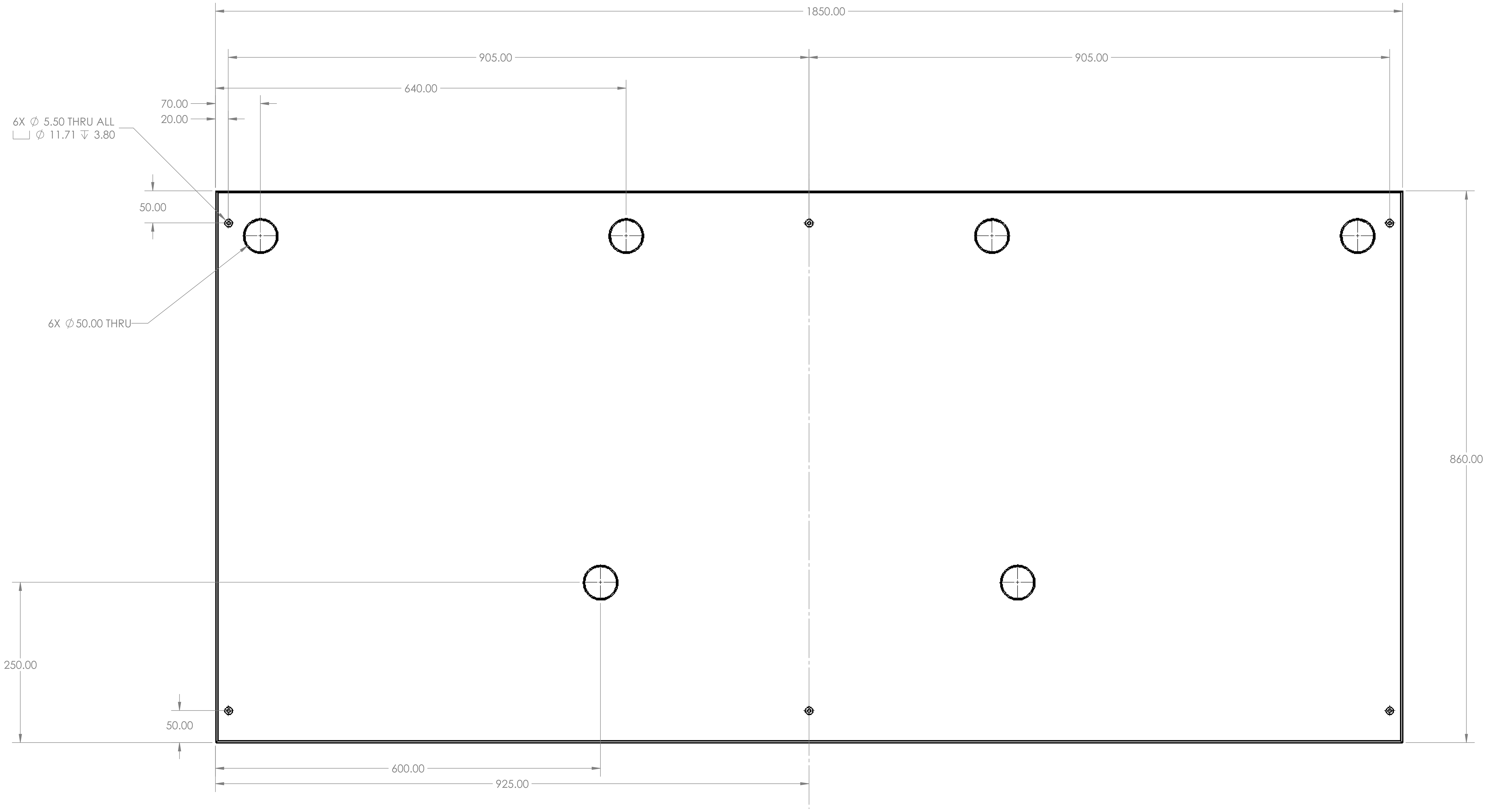

		UNLESS OTHERWISE SPECIFIED:		NAME	DATE
		DIMENSIONS ARE IN INCHES<br>TOLERANCES:<br>FRACTIONAL ±<br>ANGULAR: MACH ± BEND ±<br>TWO PLACE DECIMAL ±<br>THREE PLACE DECIMAL ±	DRAWN		
			CHECKED		
			ENG APPR.		
			MFG APPR.		
		INTERPRET GEOMETRIC TOLERANCING PER:	Q.A.		
		MATERIAL	COMMENTS:		
NEXT ASSY	USED ON	FINISH			
APPLICATION		DO NOT SCALE DRAWING			

TITLE:

SIZE DWG. NO. REV

PAL-ROBOT-ENCLOSURE-ASM_v2

SCALE: 1:10 WEIGHT: SHEET 3 OF 9

ITEM NO.	QTY.	DESCRIPTION	LENGTH
1	3	40-4040-UL	260
2	2	40-4040-UL	821.9

ITEM NO.	PART NUMBER	DESCRIPTION	QTY.
1	CHILLER-DRAWER_v2		1
		40-4040-UL	3
		40-4040-UL	2

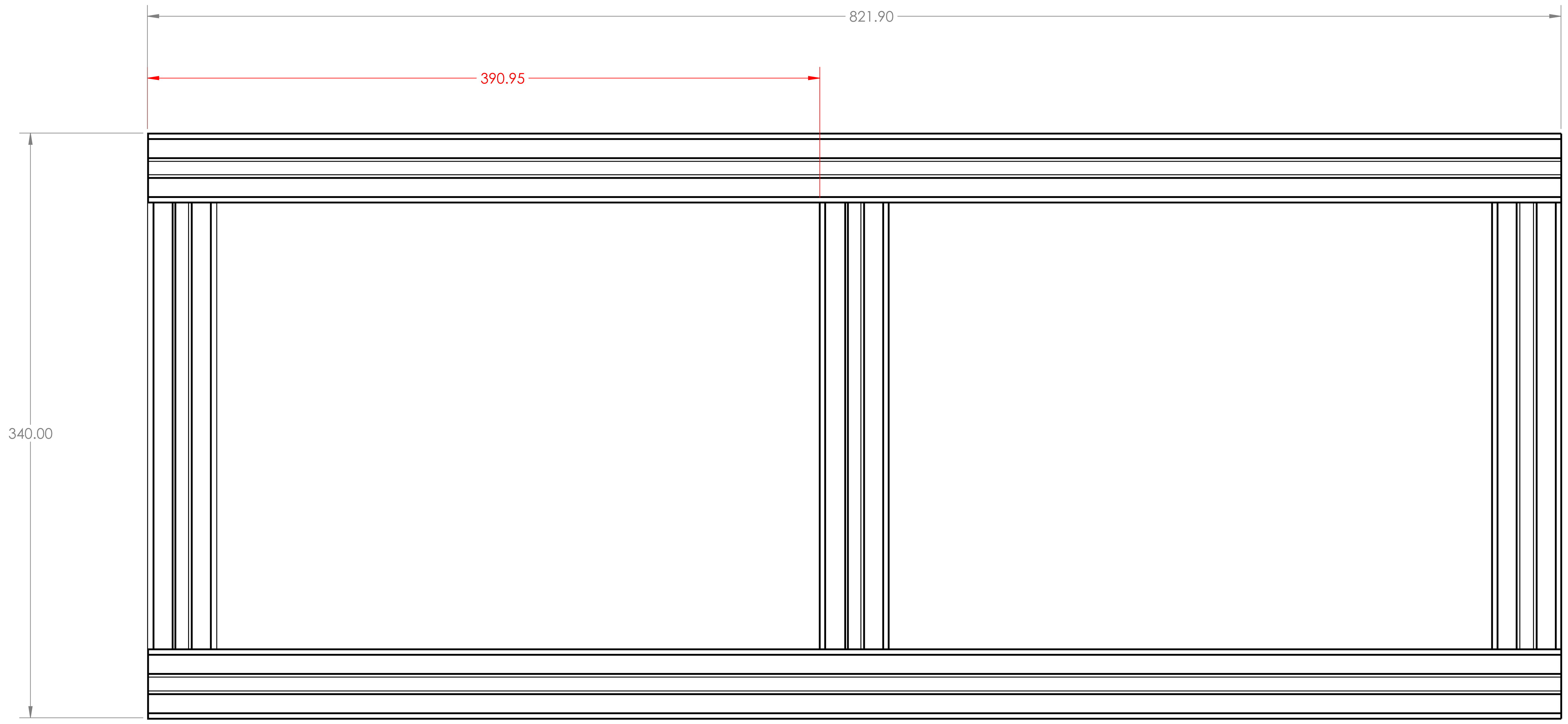

UNLESS OTHERWISE SPECIFIED:
DIMENSIONS ARE IN INCHES
TOLERANCES:
FRACTIONAL ±
ANGULAR: MACH ± BEND ±
TWO PLACE DECIMAL ±
THREE PLACE DECIMAL ±
INTERPRET GEOMETRIC TOLERANCING PER:
MATERIAL
FINISH
NEXT ASSY | USED ON
APPLICATION
DO NOT SCALE DRAWING

	NAME	DATE
DRAWN		
CHECKED		
ENG APPR.		
MFG APPR.		
Q.A.		

COMMENTS:

TITLE:

SIZE D | DWG. NO. PAL-ROBOT-ENCLOSURE-ASM_v2 | REV

SCALE: 1:5 | WEIGHT: |

NOTES:
1. 6MM POLYMETAL

ITEM NO.	PART NUMBER	DESCRIPTION	QTY.
1	CHILLER-DRAWER-SHELF_v2	POLYMETAL	1

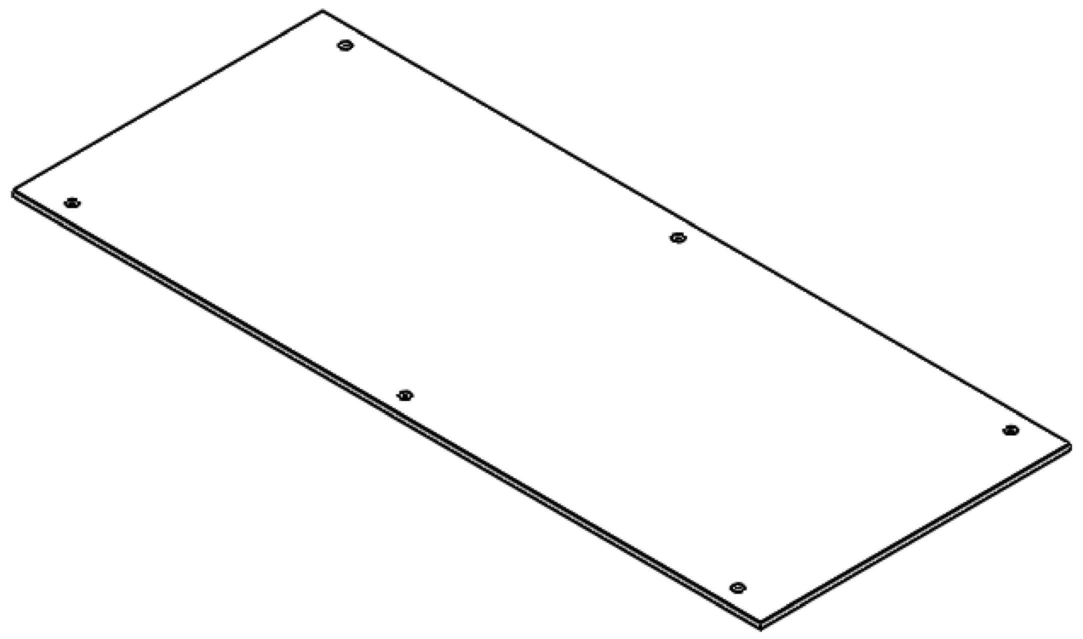

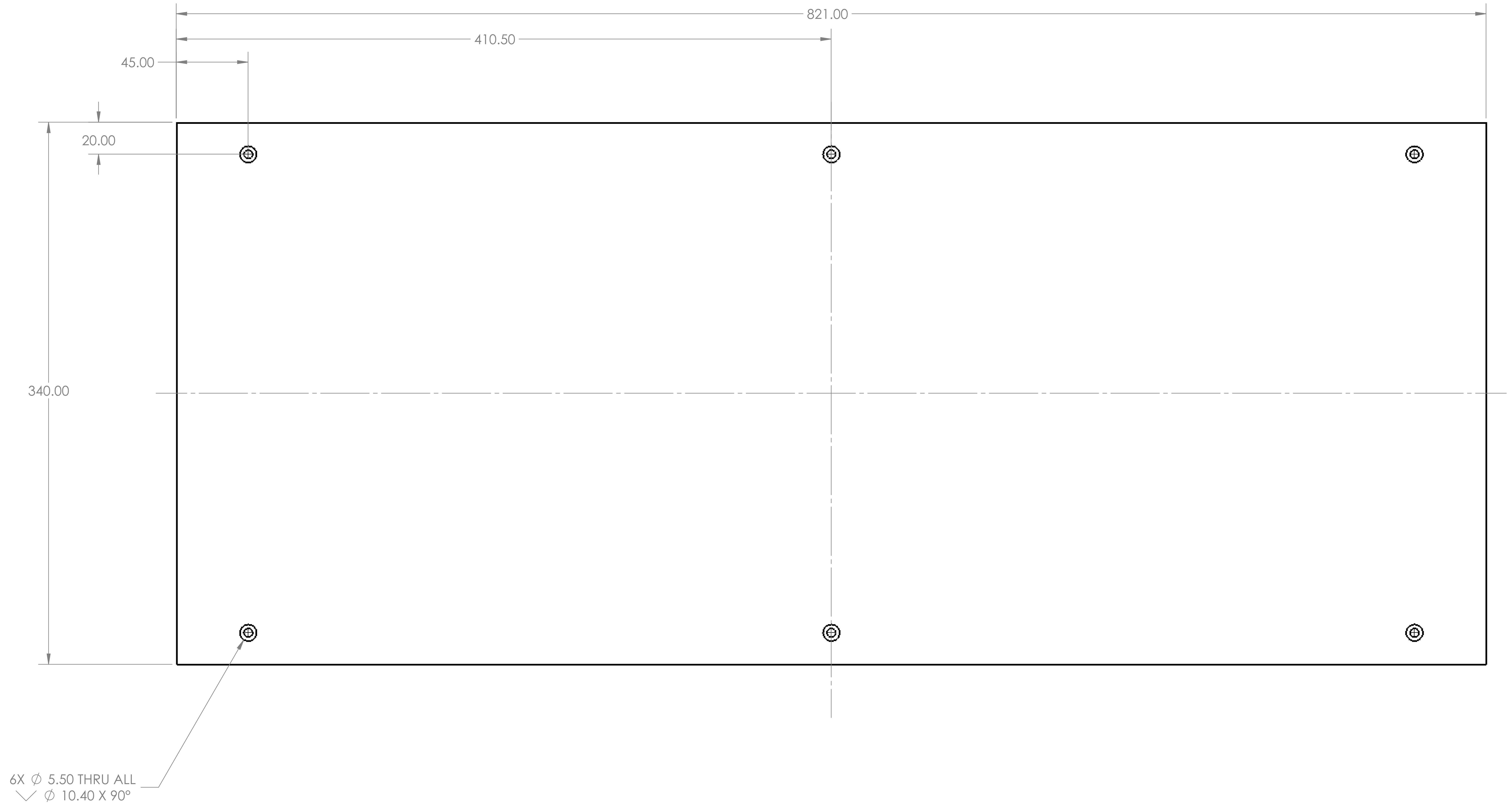

		UNLESS OTHERWISE SPECIFIED:		NAME	DATE
		DIMENSIONS ARE IN INCHES<br>TOLERANCES:<br>FRACTIONAL ±<br>ANGULAR: MACH ± BEND ±<br>TWO PLACE DECIMAL ±<br>THREE PLACE DECIMAL ±	DRAWN<br>CHECKED<br>ENG APPR.<br>MFG APPR.<br>Q.A.		
		INTERPRET GEOMETRIC TOLERANCING PER:	COMMENTS:		
		MATERIAL			
NEXT ASSY	USED ON	FINISH			
APPLICATION		DO NOT SCALE DRAWING			

TITLE:

SIZE D | DWG. NO. PAL-ROBOT-ENCLOSURE-ASM_v2 | REV

SCALE: 1:5 | WEIGHT: | SHEET 5 OF 9

ITEM NO.	PART NUMBER	DESCRIPTION	QTY.
1	BOTTOM-POLYMETAL_v2	POLYMETAL	1

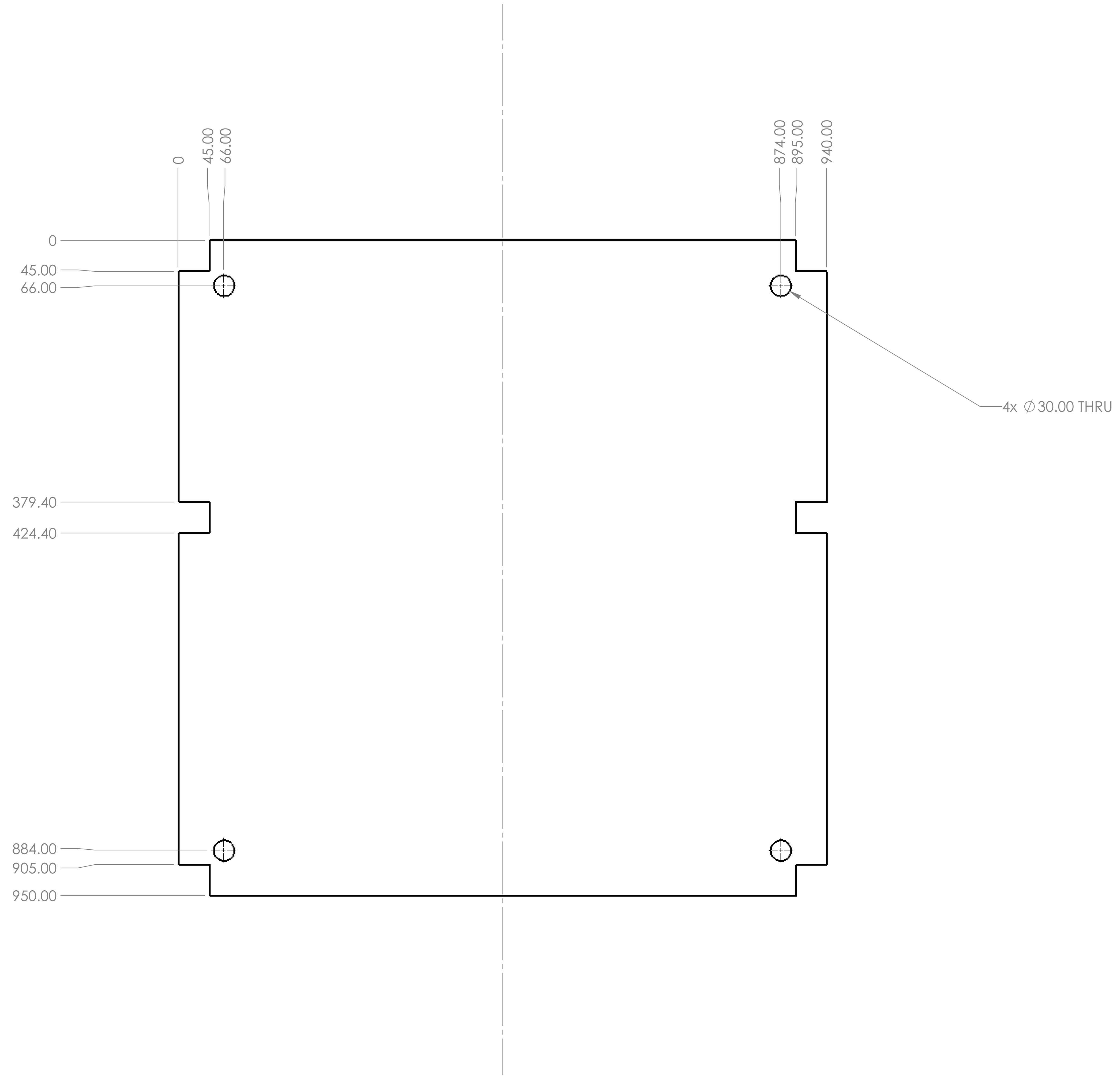

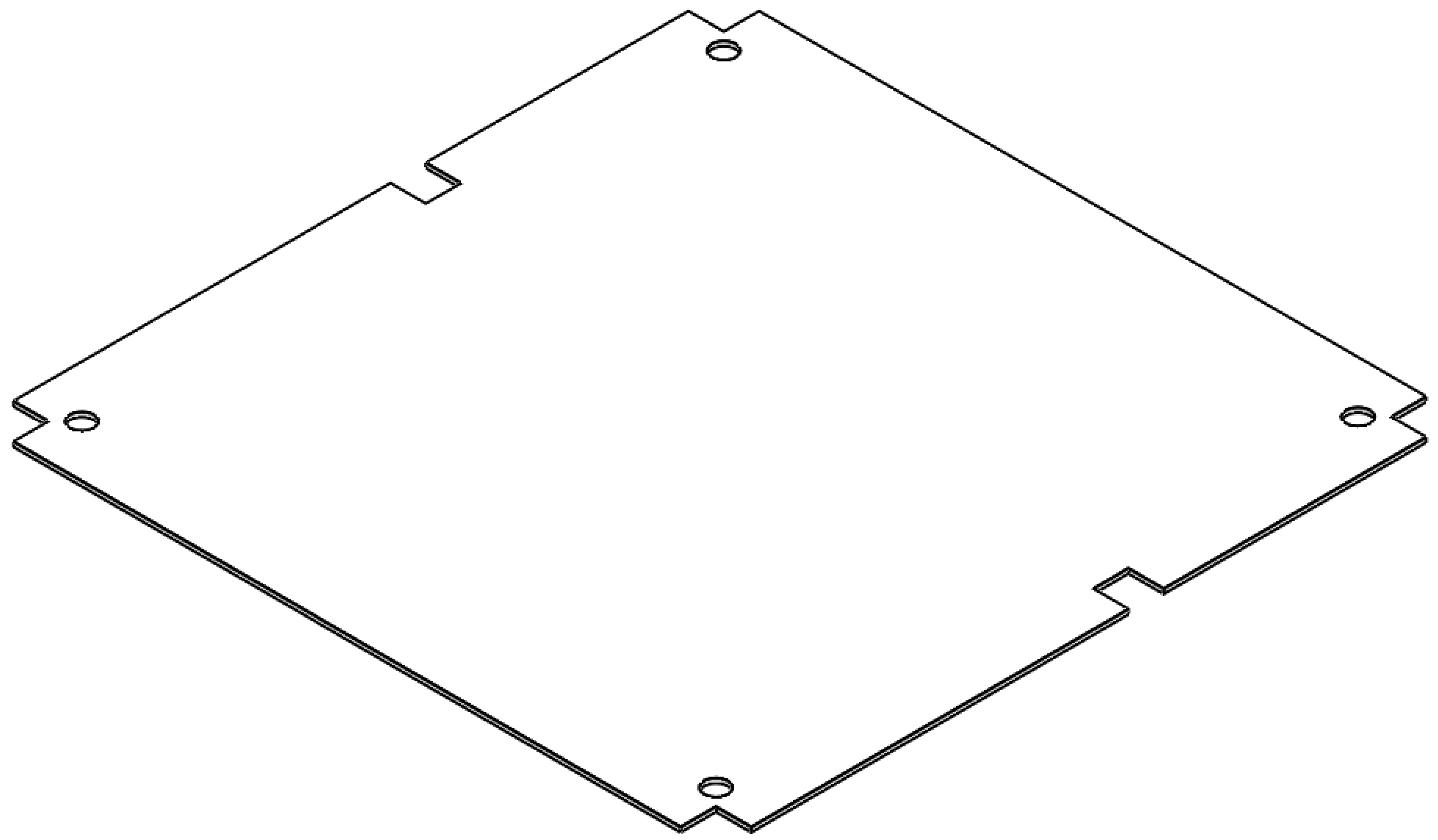

UNLESS OTHERWISE SPECIFIED:		NAME / DATE
DIMENSIONS ARE IN INCHES<br>TOLERANCES:<br>FRACTIONAL ±<br>ANGULAR: MACH ± BEND ±<br>TWO PLACE DECIMAL ±<br>THREE PLACE DECIMAL ±	DRAWN<br>CHECKED<br>ENG APPR.<br>MFG APPR.<br>Q.A.	
INTERPRET GEOMETRIC TOLERANCING PER:	COMMENTS:	TITLE:
MATERIAL		
FINISH		SIZE D / DWG. NO. PAL-ROBOT-ENCLOSURE-ASM_v2 / REV
NEXT ASSY / USED ON		
APPLICATION / DO NOT SCALE DRAWING		SCALE: 1:5 / WEIGHT: / SHEET 6 OF 9

ITEM NO.	PART NUMBER	DESCRIPTION	QTY.
1	NARROW-SHELF_v2	POLYMETAL	1

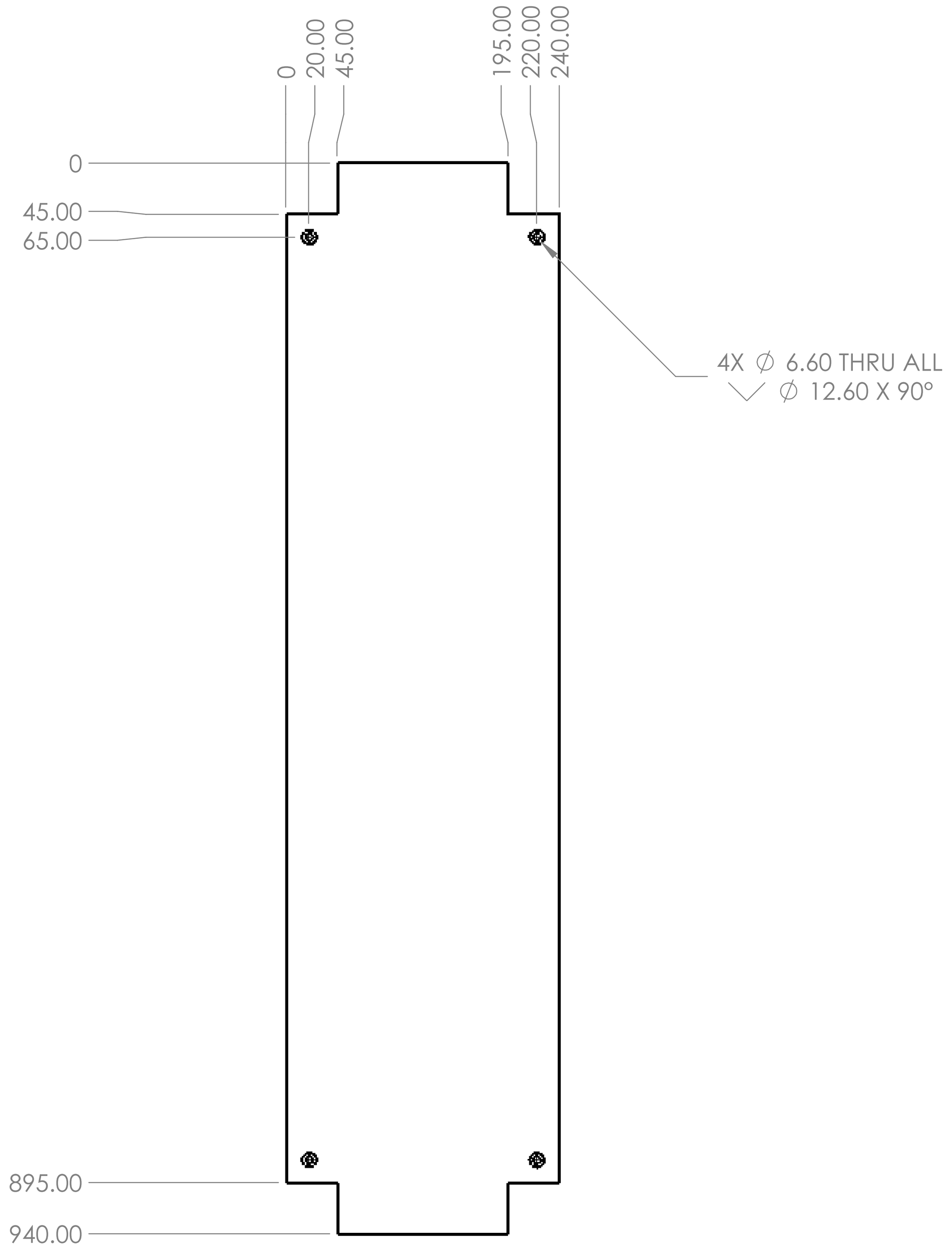

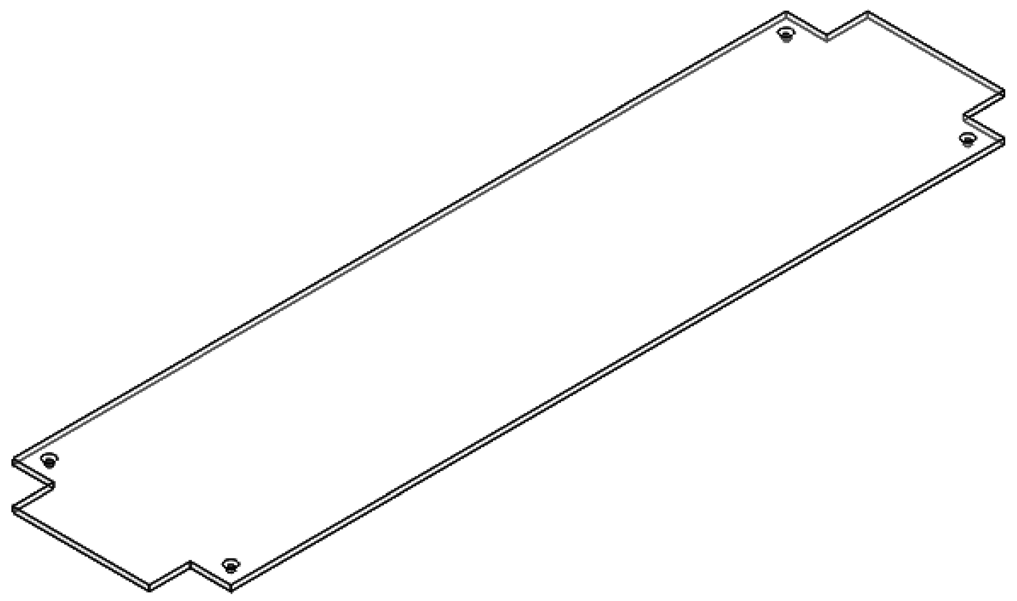

UNLESS OTHERWISE SPECIFIED:		
DIMENSIONS ARE IN INCHES<br>TOLERANCES:<br>FRACTIONAL ±<br>ANGULAR: MACH ± BEND ±<br>TWO PLACE DECIMAL ±<br>THREE PLACE DECIMAL ±		
INTERPRET GEOMETRIC TOLERANCING PER:		
MATERIAL		
FINISH	NEXT ASSY	USED ON
DO NOT SCALE DRAWING	APPLICATION	

	NAME	DATE
DRAWN		
CHECKED		
ENG APPR.		
MFG APPR.		
Q.A.		
COMMENTS:		

TITLE:

SIZE	DWG. NO.	REV
D	PAL-ROBOT-ENCLOSURE-ASM_v2	
SCALE: 1:5	WEIGHT:	SHEET 7 OF 9

ITEM NO.	PART NUMBER	DESCRIPTION	QTY.
1	COMPUTER-SURFACE_v2	POLYMETAL	1

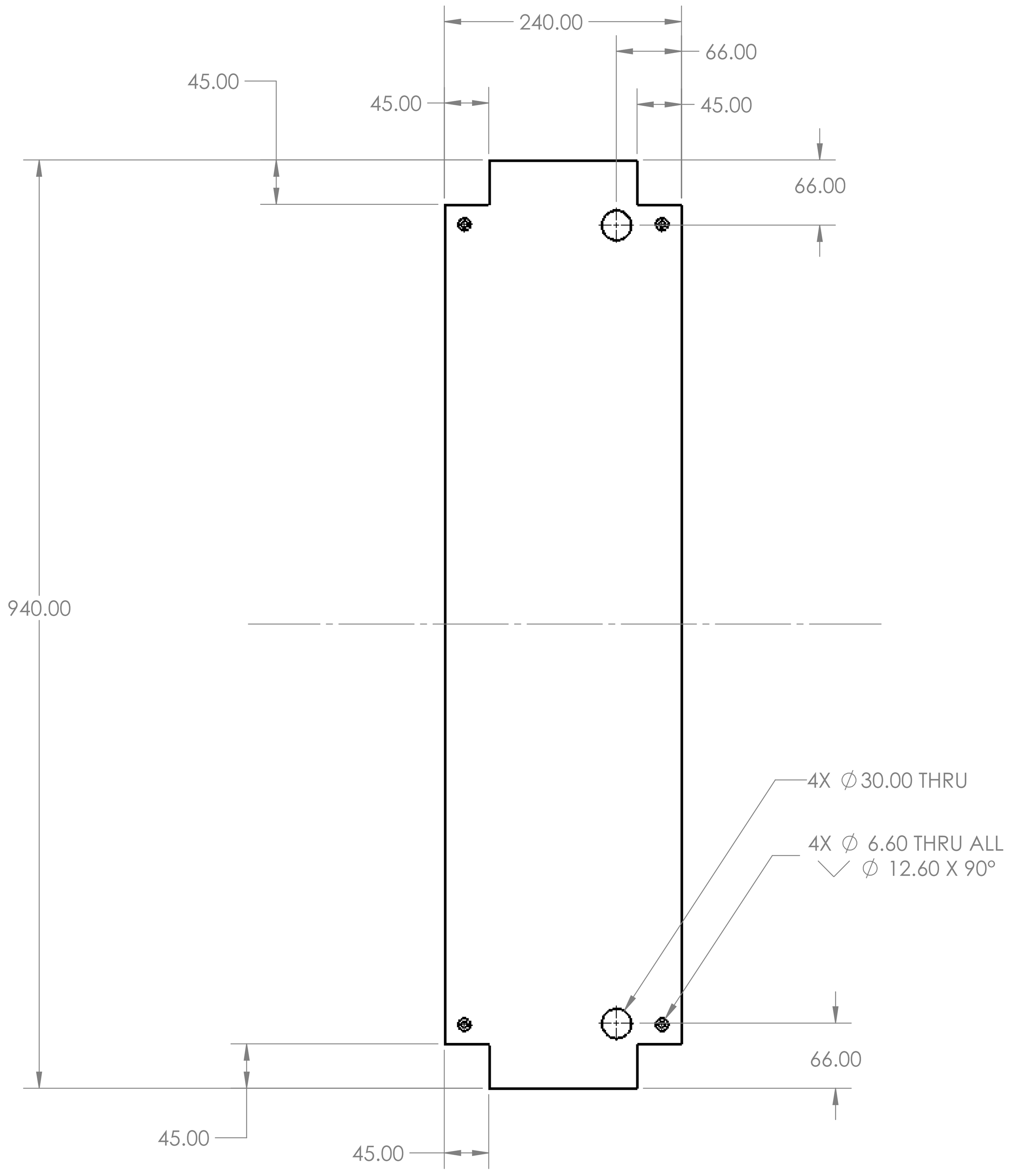

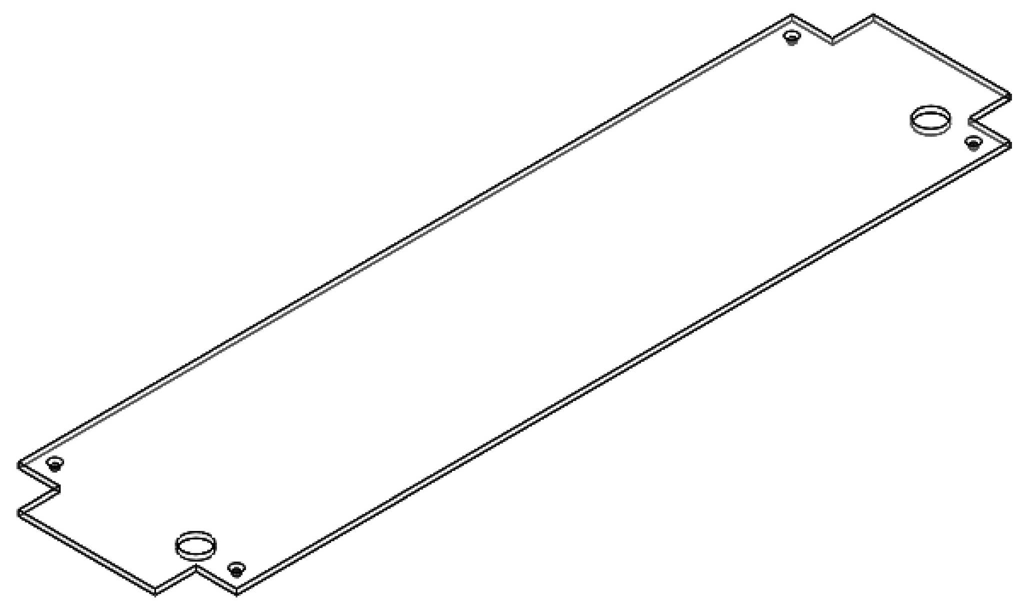

UNLESS OTHERWISE SPECIFIED:
DIMENSIONS ARE IN INCHES
TOLERANCES:
FRACTIONAL ±
ANGULAR: MACH ± BEND ±
TWO PLACE DECIMAL ±
THREE PLACE DECIMAL ±
INTERPRET GEOMETRIC TOLERANCING PER:
MATERIAL
FINISH
NEXT ASSY | USED ON
APPLICATION
DO NOT SCALE DRAWING

	NAME	DATE
DRAWN		
CHECKED		
ENG APPR.		
MFG APPR.		
Q.A.		
COMMENTS:		

TITLE:

SIZE D | DWG. NO. PAL-ROBOT-ENCLOSURE-ASM_v2 | REV

SCALE: 1:5 | WEIGHT: | SHEET 8 OF 9

ITEM NO.	PART NUMBER	DESCRIPTION	QTY.
1	TOP-PANEL_v2	Acrylic (Medium-high impact)	1

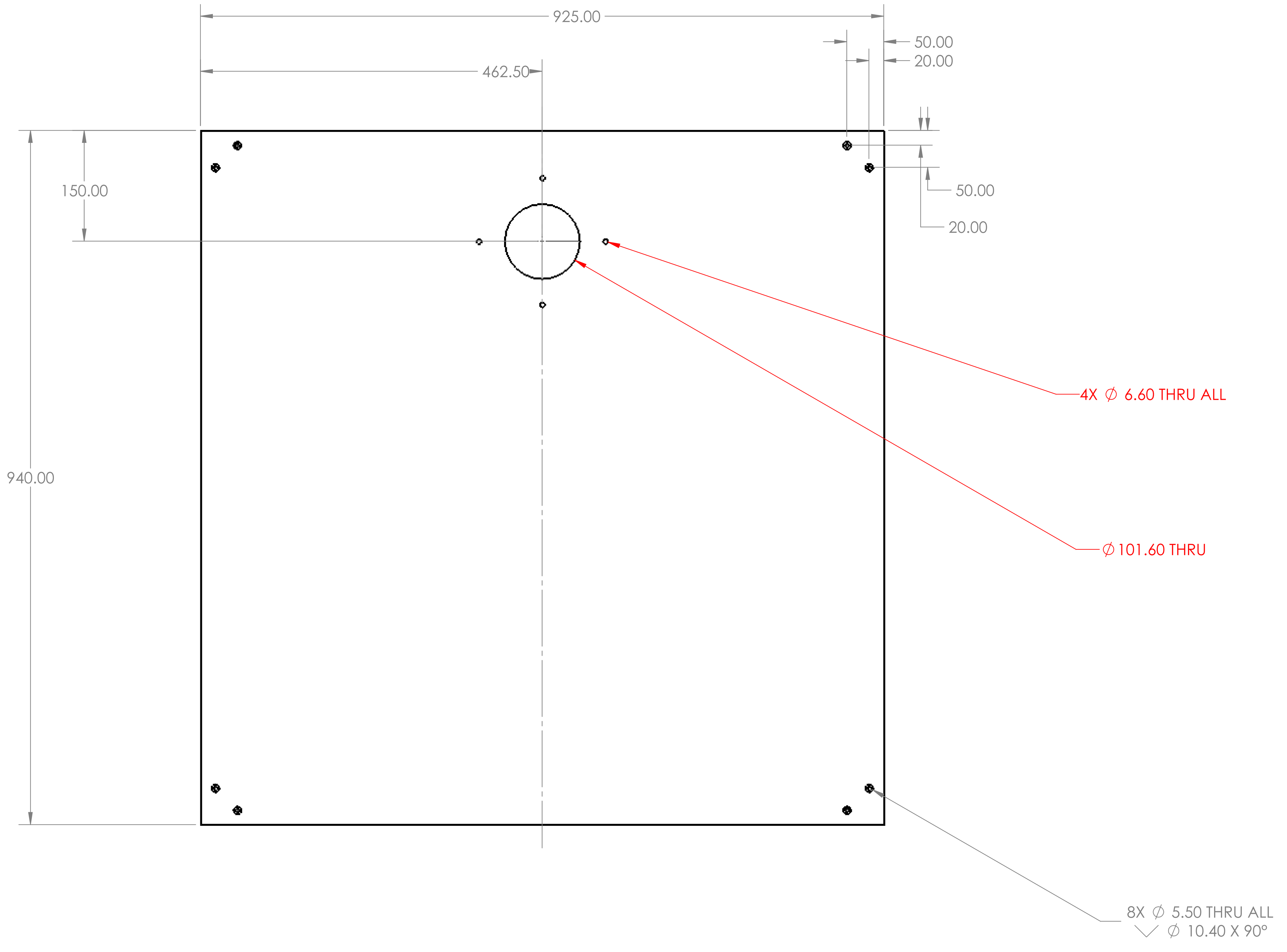

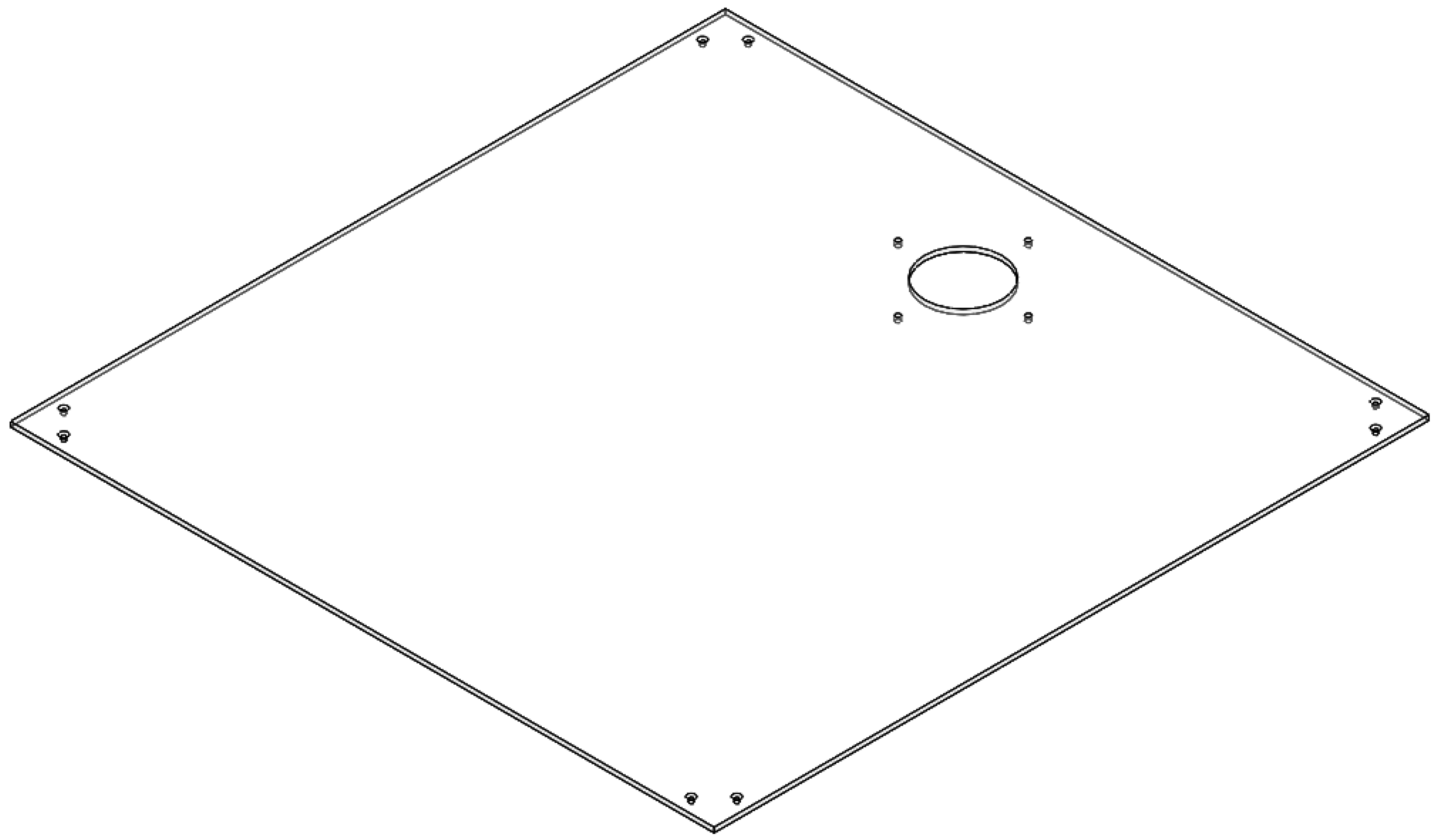

		UNLESS OTHERWISE SPECIFIED:		NAME	DATE	
		DIMENSIONS ARE IN INCHES<br>TOLERANCES:<br>FRACTIONAL ±<br>ANGULAR: MACH ± BEND ±<br>TWO PLACE DECIMAL ±<br>THREE PLACE DECIMAL ±	DRAWN			TITLE:
			CHECKED			
			ENG APPR.			
			MFG APPR.			
		INTERPRET GEOMETRIC TOLERANCING PER:	Q.A.			
		MATERIAL	COMMENTS:			SIZE D DWG. NO. PAL-ROBOT-ENCLOSURE-ASM_v2 REV
NEXT ASSY	USED ON	FINISH				
APPLICATION		DO NOT SCALE DRAWING				SCALE: 1:5 WEIGHT: SHEET 9 OF 9
